# Team Flow Is a Unique Brain State Associated with Enhanced Information Integration and Interbrain Synchrony

**DOI:** 10.1523/ENEURO.0133-21.2021

**Published:** 2021-10-12

**Authors:** Mohammad Shehata, Miao Cheng, Angus Leung, Naotsugu Tsuchiya, Daw-An Wu, Chia-huei Tseng, Shigeki Nakauchi, Shinsuke Shimojo

**Affiliations:** 1 Division of Biology and Biological Engineering, California Institute of Technology, Pasadena 91125, CA; 2 The Electronics-Inspired Interdisciplinary Research Institute (EIIRIS), Toyohashi University of Technology, Toyohashi 441-8580, Japan; 3 The University of Hong Kong, Pokfulam 999077, Hong Kong; 4 NTT Communication Science Laboratories, NTT Corporation, Atsugi 243-0198, Japan; 5 School of Psychological Sciences and Turner Institute for Brain and Mental Health, Monash University, Melbourne, Victoria 3800, Australia; 6 Center for Information and Neural Networks (CiNet), National Institute of Information and Communications Technology (NICT), Suita 565-0871, Japan; 7 Advanced Telecommunications Research Computational Neuroscience Laboratories, Kyoto 619-0288, Japan; 8 Research Institute of Electrical Communication, Tohoku University, Sendai 980-8577, Japan; 9 Department of Computer Science and Engineering, Toyohashi University of Technology, Toyohashi 441-8580, Japan

**Keywords:** EEG, flow, hyperscanning, in the zone, neural synchrony, teams

## Abstract

Team flow occurs when a group functions in a high task engagement to achieve a goal, commonly seen in performance and sports. Team flow can enable enhanced positive experiences, as compared with individual flow or regular socializing. However, the neural basis for this enhanced behavioral state remains unclear. Here, we identified neural correlates (NCs) of team flow in human participants using a music rhythm task with electroencephalogram hyperscanning. Experimental manipulations held the motor task constant while disrupting the corresponding hedonic music to interfere with the flow state or occluding the partner’s positive feedback to impede team interaction. We validated these manipulations by using psychometric ratings and an objective measure for the depth of flow experience, which uses the auditory-evoked potential (AEP) of a task-irrelevant stimulus. Spectral power analysis at both the scalp sensors and anatomic source levels revealed higher β-γ power specific to team flow in the left middle temporal cortex (L-MTC). Causal interaction analysis revealed that the L-MTC is downstream in information processing and receives information from areas encoding the flow or social states. The L-MTC significantly contributes to integrating information. Moreover, we found that team flow enhances global interbrain integrated information (II) and neural synchrony. We conclude that the NCs of team flow induce a distinct brain state. Our results suggest a neurocognitive mechanism to create this unique experience.

## Visual Abstract

**Figure:**
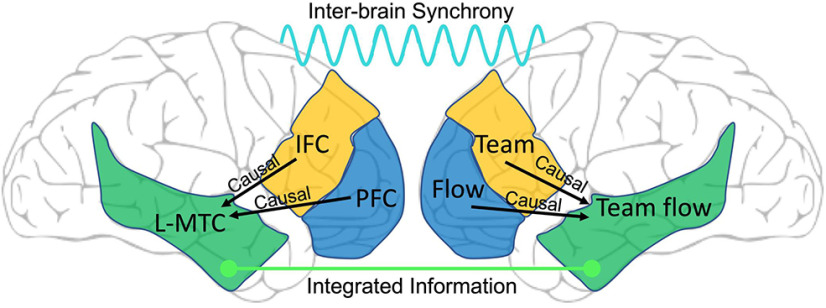

## Significance Statement

This report presents neural evidence that teams falling into the flow state (team flow), a highly positive experience, have a unique brain state distinct from ordinary flow or social states. We established a new objective neural measure of flow yet consistent with subjective reports. We identified neural markers of team flow at the left middle temporal cortex (L-MTC). We showed the L-MTC had a unique causality and contributed to information integration during team flow. Finally, we showed that team flow is an independent interbrain state with enhanced information integration and neural synchrony. The data presented here suggest a neurocognitive mechanism of team flow.

## Introduction

Flow state, or “getting into the zone,” is a psychological phenomenon that develops when balancing the performance with the challenge of a task and providing clear goals and immediate feedback (Csikszentmihalyi, 1975; Nakamura and Csikszentmihalyi, 2002). The flow state is characterized by intense task-related attention, effortless automatic action, a strong sense of control, a reduced sense of external and internal awareness, and a reduced sense of time (Nakamura and Csikszentmihalyi, 2002). The flow state is intrinsically rewarding and can positively affect subsequent experiences (Csikszentmihalyi, 1975, 2014; Nakamura and Csikszentmihalyi, 2002; Harmat, 2016). Because of these characteristics, flow is an intensely-studied topic in sports, music, education, work, and gaming. The flow state can develop during an individual (solo) activity or a group activity. There is a growing interest in studying flow in group activities, i.e., group flow, among several fields including psychology, sociology, organizational behavior, and business (Sawyer, 2007; Walker, 2010; Hart and Di Blasi, 2013; Salanova et al., 2014; Hari et al., 2015; Pels et al., 2018). Team flow is a specific case of group flow in which the group forms a team that is characterized by a common purpose, complementary skills, clear performance goals, strong commitment, and mutual accountability (Katzenbach and Smith, 1993a,b; van den Hout et al., 2018). The positive subjective experience during team flow, as in sports teams, music ensembles, dance squads, business teams, or video gaming teams, is superior to everyday social interaction or experiencing individual flow (Sato, 1988; Hari et al., 2015; Pels et al., 2018).

A simplistic assumption is that team flow is a simple combination of the flow and the social states. These two states are disparate, in other words, acting in a social context is not necessarily sufficient to get into the flow state, and vice versa. In prior reports, the neural mechanisms underlying the individual flow state and social experience have been studied in isolation. For social information processing, several networks have been implicated. Social perception, empathy, mentalization, and action observation networks may provide partially overlapping brain regions in conjunction with the amygdala, anterior cingulate cortex (ACC), prefrontal cortex (PFC), inferior frontal gyrus (IFG), and the inferior and superior parietal lobule (IPL/SPL), respectively (Ongür and Price, 2000; Dodell-Feder et al., 2011; Lamm et al., 2011; Molenberghs et al., 2012; Stanley and Adolphs, 2013; Yang et al., 2015). Meanwhile, several studies of individual flow have shown increased activity in the IFG and the IPL/SPL, and decreased activity in the PFC (Klasen et al., 2012; Ulrich et al., 2014, 2016a,b; Harris et al., 2017). We cannot hypothesize that any of the aforementioned brain regions contribute to team flow since there are concordant and discordant overlaps. Hence, we posit that team flow is more than a combination of these two states and may arise from a unique interaction among these brain regions, which would reveal new neural correlates (NCs) that create this unique team flow brain state.

Phenomenologically, the experience of team flow is subjectively more intense than the individual flow state and ordinary social state. However, the underlying neural mechanism is still unclear. This study directly examines the underlying neural activity patterns, emerging at both the intrabrain and interbrains levels during team flow. Using an exploratory approach, we identified the intrabrain correlates in team flow that are distinct from ordinary flow or social experiences. Using causality analysis, integrated information (II), and neural synchrony data, we propose a model of the neural mechanisms that underlie team flow.

## Materials and Methods

### Participants

We recruited 78 participants for the screening process. In the main EEG experiment, 15 participants (five males; age: 18–35 years) attended and formed 10 pairs (three male pairs), of which five participants (one male) were paired twice. Written informed consent was acquired from all participants. Human subjects were recruited at a location which will be identified if the article is published. All the procedures were approved by the Institutional Review Board of California Institute of Technology.

### Task

**Figure 1.**
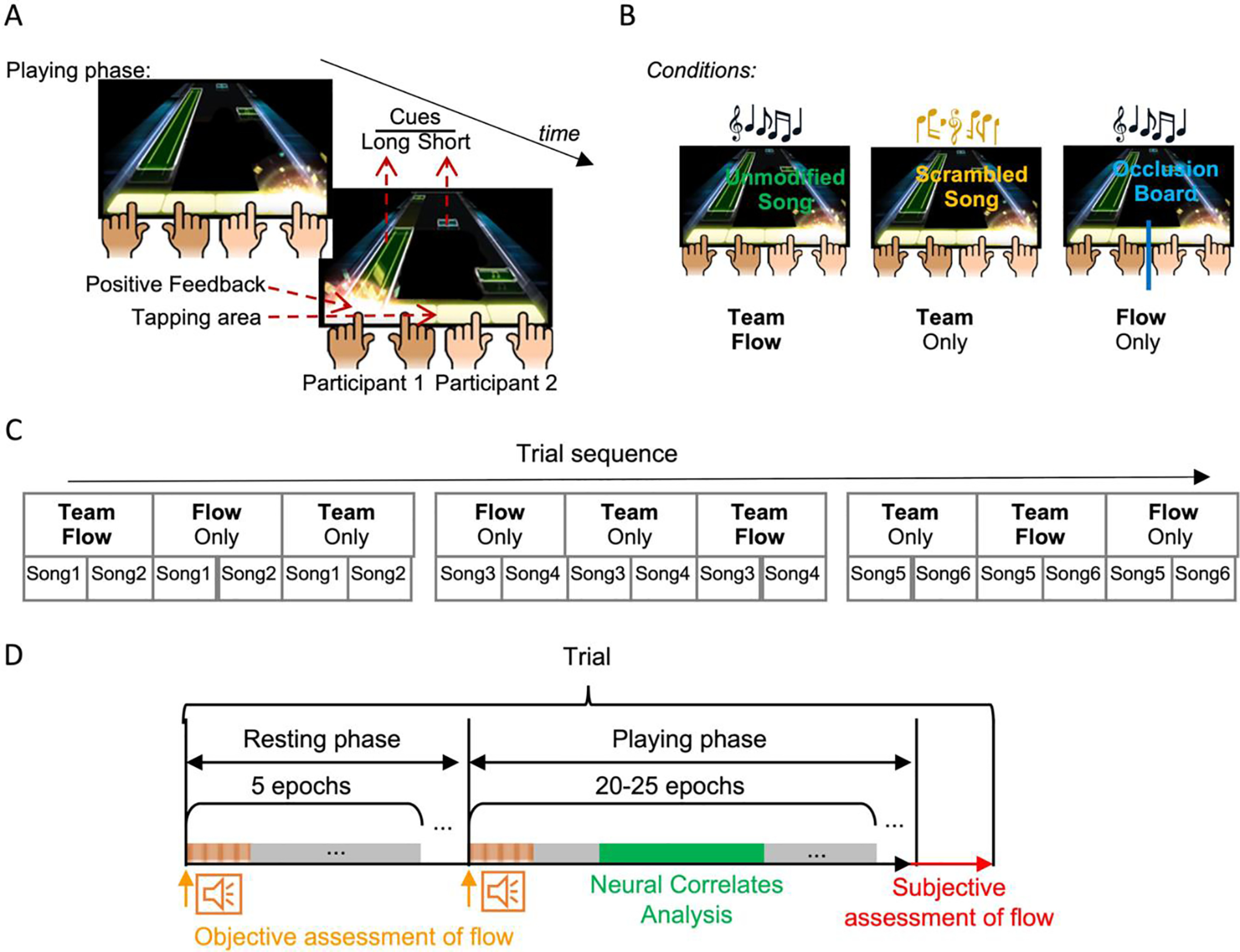
Behavioral establishment of team flow. ***A***, Diagram of the finger-tapping music rhythm game. Participants must tap when animated cues moving from the top of the screen reach the tapping area. ***B***, Manipulations: team flow is predicted when the participants are playing the unmodified song and they can see the partner’s positive feedback (Team Flow). The flow state is disrupted through scrambling the music (Team Only). Team interaction is disrupted by hiding the partner’s positive feedback using an occlusion board (Flow Only). See Table 1 for details. ***C***, Sequence of the trials, showing which song and condition per trial was assigned during the main experiment. ***D***, Trial analysis: participants were sitting still while listening to a background music during the resting phase and played the game in the playing phase. The electroencephalogram was epoched for objective assessment of flow i.e., the AEP analysis of the task-irrelevant beeps (orange bar) and for the NCs analysis (green bar). After each trial, participants answered the questionnaire for the subjective assessment of flow. Extended Data Figure 1-1 shows detailed analysis pipeline.

We used a commercial music rhythm game called “O2JAM U” (version 1.6.0.11, MOMO Co) played on an iPad air (model No. MD786LL/B, system, iOS 10.3.2). The basic structure of this game follows the most common structure in the music rhythm genre. Two consecutive screenshots of the game are shown in Figure 1*A*. Visual cues (notes) moves in lanes from the top to the bottom of the screen where the tapping area is located. There are two kinds of cues: short and long ones. A player’s task is to tap when a short cue reaches the tapping area, and to tap and hold for the duration a long cue is at the tapping area. The cues are designed to give the impression of playing a musical instrument, which produces much of the positive experience of the game. The game displays two types of real-time feedback on the players’ performance. The first feedback type includes a semantic judgment expression (“EXCELLENT,” “GOOD,” or “MISS”) together with a numerical score presented at the center and the top corners of the screen (Extended Data Fig. 1-1*A*). We made the first feedback type invisible to the participants, using a privacy screen protector, to enhance participants’ focus on the tapping area. The second feedback type is a flashing visual effect that appears at the tapping area each time the player taps at the correct timing with the cue (Extended Data Fig. 1-1*A*). We kept this feedback type visible to the participants as positive reinforcement (Movie 1). The game provides two modes of play: a two-lane or four-lane mode, in which either two or four lanes of moving cues are presented. We used the two-lane mode during individual screening with the participant responsible for both lanes. We used the four-lane mode during the main experiment in which a pair of participants played with each participant responsible for two adjacent lanes.

10.1523/ENEURO.0133-21.2021.f1-1 Extended Data Figure 1-1 Experimental design. ***A***, A screenshot of the game showing feedback and position of the card board. The first type of feedback was invisible to participants. The second type of feedback was visible to participants in the Inter-SyncA and Inter-ScrA conditions. The cardboard (red dashed line) occluded the tapping area, the second type of feedback, and the whole bodies of participants including fingers. The cardboard kept the visual cues visible to participants (green dashed rectangle). ***B***, Trial and analyses details: participants were sitting still while listening to a background music during the resting phase. The electroencephalogram was epoched for the AEP analysis of the task-irrelevant beep sound (AEP epochs; orange) and for the NCs analysis (NC epochs; green). All NC analyses, with the corresponding figure or table, are summarized in steps 3–6. Download Figure 1-1, TIF file.

**Table 1.** Comparison of the stimuli across the experimental conditions

Conditions		Team Flow	Team Only	Flow Only
Cues sequence (visual stimulus)	Self	Constant (visible to both participants)		
	Partner	Constant (visible to both participants)		
Positive Feedback	Self	Visible (performance dependent)		
	Partner	Visible	Visible	*Not visible*
Song (auditory stimulus)		Original	*Scrambled*	Original
Beeps (task-irrelevant stimulus)		Constant	Constant	Constant

Movie 1. A few seconds of game-play in the Team Flow and Team Only conditions. The “beeps” word at the bottom right indicates the timing of the task-irrelevant beep sound presentation. These words are overlaid in the video for illustration and were not present during the experiment. The scores and other indicators at the center and at the top right and left corners were hidden from the participants. 10.1523/ENEURO.0133-21.2021.video.1

After playing each song (trial), the game displays a performance report on the screen, including a final numerical score, the total number of cues, and the number of missed cues. The performance report of each trial was hidden from the participants until they finish answering their subjective experience psychometric ratings. The percentage of the missed cues per the total number of cues was used as a metric for the performance of each pair of participants.

### Manipulations

To create the team flow condition, the iPad was tilted and positioned, using a custom-made holder, equidistant from the pair of participants. Participants were instructed to sit on two chairs at a fixed distance, to keep their heads on chin rests, and to minimize their body movements except for finger movement. The iPad was connected to a pair of stereo speakers placed horizontally and equidistant from the iPad and the pair of participants. A pair of participants played in the four-lane mode.

Previous studies controlled the flow experience by manipulating the skill-challenge level in the experimental setup, either passively by free task performance and retrograde classification of certain time periods into several flow levels (Klasen et al., 2012) or by actively controlling the task level to be either too easy (boredom), adaptive (flow), or too hard (overload; Ulrich et al., 2014, 2016a,b). One of the issues with modifying the skill-challenge level to study flow is that this changes other cognitive functions, such as attention, sensory information processing, and cognitive load necessary to perform each task, as well as gross changes in motor behavior. Therefore, manipulating the skill-challenge level complicates the ability to distinguish the neural mechanisms underlying team flow interaction. To avoid this complexity, we kept the task identical in all conditions using the same sequence of tapping cues. We manipulated the intrinsic reward/enjoyment dimension of flow by scrambling the game music and hence disrupting the pleasant experience (Table 1).

To create the team only condition, participants played the same song (i.e., an identical sequence of moving cues) as the team flow using the same setup; however, a reversed and shuffled version of the music was played from the same speakers (Table 1). The music for each song was reversed through an online audio editing website (https://audiotrimmer.com/online-mp3-reverser/) and then cut into 5-s fragments through an online audio cutter (http://mp3cut.net/). We randomly shuffled the fragments and rejoined them through an online audio joiner (http://audio-joiner.com/).

To create the flow only condition, participants played the same song (i.e., an identical sequence of moving cues) as the team flow using the same setup; however, a black foam board (1 cm × 1.5 m × 75 cm) was placed between the chairs to completely block the participants’ view of each other, and a black piece of cardboard was placed across the iPad screen with an opening to show the visual cues but not the tapping area (Extended Data Fig. 1-1*A*).

### Screening process

The participants were first tested using a selected song (269 cues per the two lanes) in the two-lane mode to exclude unexperienced participants. Participants were qualified to complete the study if they missed no more than 10 cues. Out of the 78 participants recruited for the first screening test, 54 participants were qualified and the remaining 24 participants were excluded. The 54 qualified participants were also tested using other selected songs with a higher number of cues to confirm their skill level. Because the positive experience of this game depends on individual preference for the music rhythm, we prepared 11 songs (500–960 cues per the four lanes) in various genres. The 54 qualified participants were asked to rate their preferences for all the 11 songs separately on a seven-point scale (one for “not like it at all,” seven for “like very much”). Although the duration of the songs varied from 110 to 160 s, each song was played only for 1 min, which was long enough to ensure that participants had heard the main rhythm of the song. We fixed the duration for all songs to ensure the accuracy of preference ratings. To avoid possible influence from the experimenter, the experimenter maintained a neutral attitude by avoiding eye contact with participants or a physical response to the music. Participants were paired based on their skill level and song preference rating.

The second step of the process was to screen paired participants for their preference to the team set-up (team flow) versus playing in the team with board set-up (flow only) or a single-player set-up (solo flow) in a behavior pilot experiment. For the solo flow condition, we used the two-lane mode of the game. At the end of the pilot experiment, we presented the following question: “Based on how much you enjoyed the performance and you want to play it again, please rank the following experiences: single-player set-up, team set-up, the team with board set-up.” We presented a scale from 1 (least preferred) to 7 (strongly preferred) in front of each set-up, and the ranking was not a forced-choice one. We excluded participants who ranked the team with board set-up or single-player set-up higher than the team set-up. Out of the 54 participants from the first screening step, 38 prosocial participants were invited for the main experiment on another day, and the remaining 24 participants were excluded. For both the screening and the main experiment, we paired only same gender participants, and the main pairing criteria for qualified participants were their skill level and song preferences. As friends were encouraged to pair up to participate in this experiment, we preferred pairing friends into the main experiment if they satisfied the main criteria. Participants reported their relation with the partner by answering the question “Generally, how much time do you spend with the other player?” Pairs who answered “first time to meet” or “only meet in the last experiment” were categorized as strangers; pairs who answered “less than 5 h per week,” “5–20 h per week,” or “more than 20 h per week” were categorized as friends. In total, 17 participants consisted of six pairs of strangers and five pairs of friends.

### Main experiment

After setting the EEG cap, the electrode positions were co-registered with the T1-magnetic resonance imaging (MRI) using the Brainsight TMS Navigation system (Rogue Resolutions Ltd). Then the paired participants, seated on two chairs at a fixed distance, underwent a beep-only trial. In this trial, they were instructed to passively listen to the task-irrelevant beep stimulus for 2.5 min, to keep their heads on chin rests and their eyes open, and to minimize their body movements. This trial was to check the EEG recording quality and verify that we could obtain a clear AEP response. The paired participants then performed a practice trial in the flow only condition to become familiarized with the procedure. Then each pair of participants was required to play six songs each at the team flow, team only, or flow only conditions forming 18 trials (Fig. 1*C*,*D*). One pair played only five songs because of time availability. The sequence of songs and conditions were pseudorandomized (Fig. 1*D*). To keep participants’ continuous interest, the consecutive songs were always different (Fig. 1*D*). To control practice and carryover effects, we arranged each condition to have an equal chance of being before or after the other two conditions (Fig. 1*D*). All trials included a resting phase and a playing phase (Fig. 1*C*). During the resting phase, participants were instructed to passively listen to the task-irrelevant beep sound and the game background music for 30 s, to keep their heads on chin rests and their eyes open, and to minimize their body movements. Then, the experimenter asked participants to click the game-play icon on the iPad to start the playing phase.

During the playing phase, participants were instructed to keep their heads on chin rests, to minimize their body movements except for finger movement, and to minimize vocal sounds that could distract their partner. The participants were allowed to give verbal comments related to the game. The participants did not comment while playing the game. They were only allowed to give verbal comments after answering the psychometric ratings and revealing the participants’ final scores. We video recorded a top-view of the iPad and the participants’ hands using an iPhone fixed ∼50 cm above the iPad where all the types of feedbacks were visible. After the playing phase of each trial, participants were given access to private screens and keyboards to freely answer the psychometric ratings on the flow experience and team interaction experience. Then, the pair were allowed to jointly view the performance report. The experimenter asked the participants whether they wanted to proceed to the next trial or if they needed some rest to minimize the effect of fatigue on performance or EEG recording quality.

### Task-irrelevant stimulus

A task-irrelevant auditory stimulus (a beep sound) was pseudorandomly presented to probe the strength of the participants’ selective attention to the game and was used as an objective measure of flow. We presented beep trains played at 5 Hz for 1 s (i.e., each train consisted of five beeps). Each beep was at 500 Hz and lasted for 10 ms. The beep trains simulated the sound of someone knocking on a door to make the stimulus as natural as possible. The interval between the beep train varied from 4 to 8 s. The beeps were generated by MATLAB 2012 (The MathWorks) and delivered through another pair of speakers placed equidistant from the iPad.

### Anatomical MRI acquisition

To increase the accuracy of source estimation for cortical activity, individual head anatomy from each participant, who passed the screening and agreed to participate in the main experiment, was acquired with MRI. A 3 Tesla Siemens Trio scanner and standard radio frequency coil was used for the entire MRI scanning. High resolution structural images were collected using a standard MPRAGE pulse sequence, providing full brain T1-weighted 3D structural images.

### Psychometric ratings and calculation of experience indices

From subjective reports, we calculated the flow, team, and team flow indices, by calculating the arithmetic mean of the ratings for each trial, to estimate subjective experience for flow state, positive team interaction, and team flow, respectively (Extended Data Fig. 2-1). For assessing flow experience, we used psychometric ratings related to the skill-demand balance (Q1 and Q2), feeling in control (Q3), automaticity (Q4), enjoyment (Q5), and time perception (Q6) dimensions of flow (Nakamura and Csikszentmihalyi, 2002). For assessing team interaction, we used psychometric ratings related to awareness of partner (Q7), teamwork (Q8), and coordination (Q9) dimensions of positive team interaction. Psychometric ratings assessing competition (Q10) and distraction (Q11) were used to confirm the absence of negative team interactions and were not included in any index. The team flow index was calculated by averaging the flow and team indices. In addition, we tested the effect of friendship between the two players (friend or stranger) on the subjective rating of flow. There was no significant difference between friend-pairs and stranger-pairs in flow index, team index or team flow index (two-way repeated measures ANOVA, main effect of relation for flow index, *F*_(1,18)_ = 0.6853, *p* = 0.4186; for team index, *F*_(1,18)_ = 0.1557, *p* = 0.6978; for team flow index, *F*_(1,18)_ = 1.4992, *p* = 0.2366). Therefore, we combined friend-pairs and stranger-pairs in the following analysis.

### Hyperscanning EEG recording and preprocessing

Electroencephalogram (EEG) was recorded simultaneously from both participants using a dual BioSemi ActiveTwo system (BioSemi Inc.). Each participant wore a cap holding 128 scalp Ag/AgCl electrodes. Signals were amplified by two daisy-chained ActiveTwo AD boxes where one AD box was connected to the control PC and worked as a master controlling the other AD box to ensure synchronization. Electrode impedance was kept below 10 kΩ. For each cap, an active common mode sense (CMS) electrode and a passive driven right leg (DRL) electrode positioned near the vertex served as the ground electrodes. EEG signals were recorded at a sampling rate of 2048 Hz (later down-sampled to 256 Hz). During recording, the A1 electrode, or A2 electrode in three participants served as a reference. In the ABC layout (a Biosemi designed equiradial system), these electrodes overlap with the Cz location of the international 10–20 system. Signals were recorded and saved using ActiView/LabView software (version 8.04, BioSemi Inc.) installed on the control PC. Another master PC was used to generate the task-irrelevant beep sound and to send signals to the EEG data receiver marking the onset of each beep train (event triggers). The event triggers were used to align the EEG data with the resting and the playing phases by using a real-time projection of the top-view video recording to the control PC. The experimenter confirmed that all the onsets of the beep trains happened during the resting or the playing phase periods.

To analyze the auditory-evoked potentiation (AEP), EEG data were epoched −0.5–1 s (1.5-s total) flanking the beep train onsets (AEP epochs). To analyze the NCs of game play experience, EEG data were epoched 2–5 s (3-s total) after the beep train onset (NC epochs; Fig. 1*C*). EEG data were bandpass filtered at 0.5–50 Hz, using the Parks-McClellan FIR filter, and re-referenced to the average of all channels. After this initial preprocessing, we did a visual inspection for artifacts, including EMG, then performed artifact-rejection using automatic independent component analysis (ICA) rejection using the FASTER toolbox (Nolan et al., 2010). Bad channels showing line noise noted during recording sessions were rejected and interpolated during the FASTER preprocessing.

### Auditory-evoked potential (AEP) analysis

**Figure 2.**
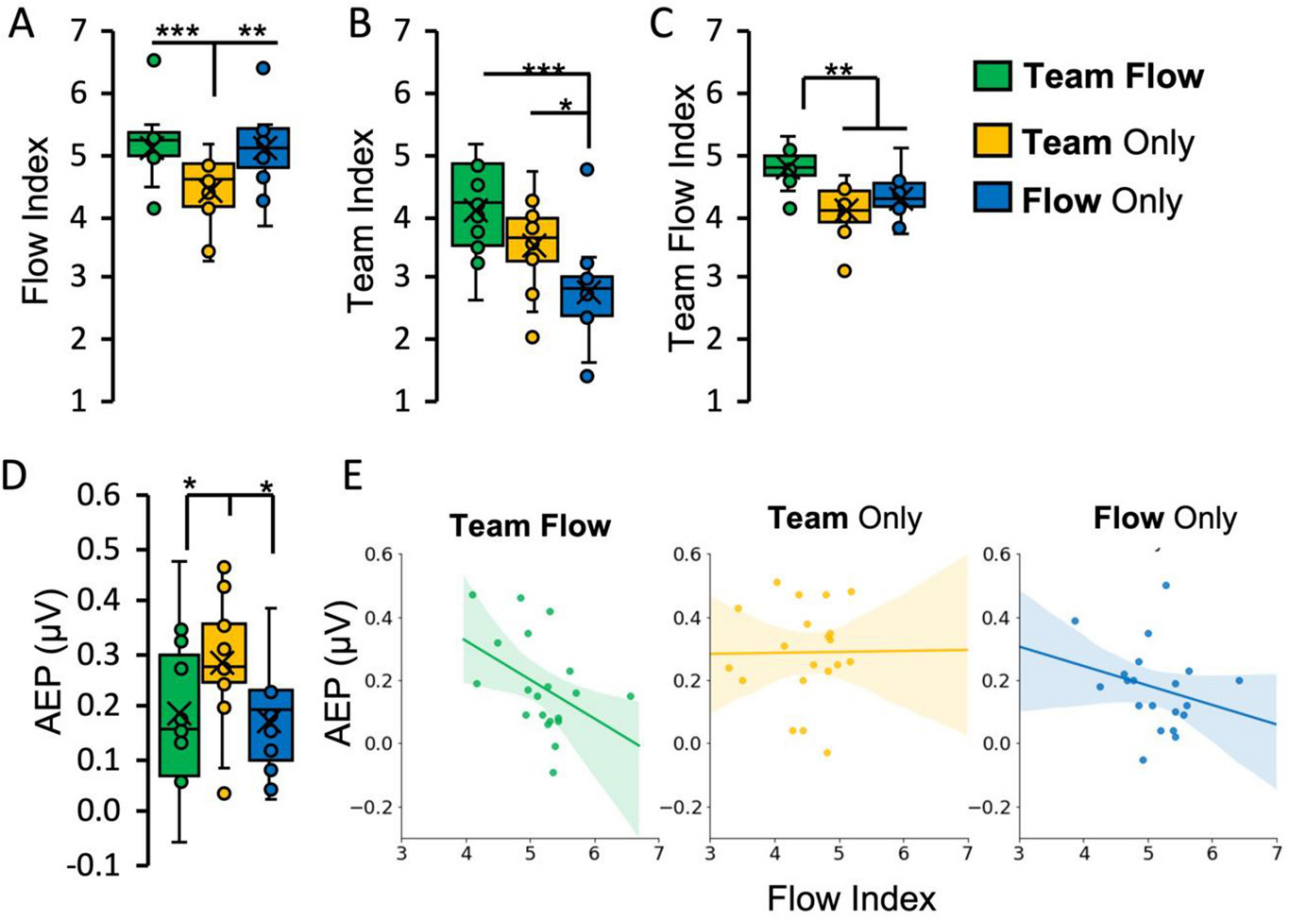
Assessment of the flow state. ***A–C***, Subjective assessment of flow: psychometric rating indices as a measure of subjective flow (flow index; ***A***), team interaction (team index; ***B***), or team flow (team flow index; ***C***) experiences (Extended Data Fig. 2-1 shows the detailed psychometric ratings for each question). Friedman test with Conover’s *post hoc* test; *p < 0.05, **p < 0.01, ***p < 0.001. Error bars represent mean ± SEM; *n* = 15. ***D***, ***E***, Objective assessment of flow. ***D***, The mean AEP calculated by averaging the following time windows: N1 (110–150 ms), P1 (210–250 ms), and N2 (310–350 ms), considering polarity. The non-flow condition (Team Only) showed statistically significant higher AEP than the flow conditions. One-way repeated measures ANOVA with Bonferroni *post hoc* test; *p < 0.05. Error bars represent mean ± SEM; *n* = 15. ***E***, Spearman’s correlation between AEP and flow index. AEP is negatively correlated with the flow index in the team flow condition (Spearman’s Rho = −0.48, *p* = 0.03), showing a negative correlation trend in the flow only condition (Spearman’s Rho = −0.29, *p* = 0.22), and no correlation in the team only condition (Spearman’s Rho = 0.11, *p* = 0.64). The lines indicate the regression lines. Shaded areas indicate a 95% confidence interval; *n* = 20. Extended Data Figure 2-2 shows the detailed AEP analysis.

To select the channels maximally responsive to the task-irrelevant auditory stimuli, we analyzed the AEP epochs during the resting phase. We calculated the event-related spectral perturbation (ERSP) and the interepoch coherence (IEC) using the EEGLAB toolbox (version 14.1.1; Brunner et al., 2013). Both ERSP and IEC showed changes in θ activity (3–7 Hz) at 100–350 ms postonset, with a peak increase at 150–250 ms postonset (Extended Data Fig. 2-2*A*,*B*). Topographical analysis in the θ band showed strong positive activity in the 14 central channels from 200–260 ms postonset (Extended Data Fig. 2-2*C*). The frequency, time, and topographical frames of our AEP were consistent with previous reports (van Driel et al., 2014; Stropahl et al., 2018). For each trial, we used IEC in the θ band during the resting phase to select channels showing stable AEP. IEC was averaged across the 14 central channels, and channels showing IEC lower than one standard error below the mean were excluded from further AEP analysis for that trial. We then analyzed θ power from −200 to 500 ms flanking the beep train onsets during the resting and the playing phase (Extended Data Fig. 2-2*A*,*B*). AEP peak amplitude was calculated according to the method described by a previous simulation report showing that event-related potential measured based on the mean amplitude surrounding the group latency is the most robust against background noise (Clayson et al., 2013). Therefore, we calculated the N1, P2, and N2 peak latencies averaged across all conditions during the resting phase (Extended Data Fig. 2-2*D*). The individual N1, P2, and N2 mean peak amplitudes ±40 ms surrounding the calculated peak latencies were obtained during the playing phase (Extended Data Fig. 2-2*E*). This resulted in the following time windows: N1 (110–150 ms), P1 (210–250 ms), and N2 (310–350 ms). The amplitude peaks at these time windows were averaged, considering polarity [i.e., (P2-N1-N2)/3], and used as AEP (Fig. 2*F*,*G*).

10.1523/ENEURO.0133-21.2021.f2-1 Extended Data Figure 2-1 Summary of subjective ratings for assessing the flow state and team interaction. The flow index was calculated by averaging responses for 1–6, the team index was calculated by averaging 7–9, and the team flow index was calculated by averaging 1–9. Psychometric ratings description: (1) “I had the necessary skill to play this trial successfully”; (2) “I will enjoy this trial more if it has less/more notes”; (3) “I felt in control while playing this trial”; (4) “I made correct movements automatically without thinking”; (5) “I love the feeling of this trial and want to play it again”; (6) “How time flies during this trial”; (7) “I was aware of the other player’s actions”; (8) “I felt like I was playing with the other person as a team”; (9) “I was coordinating my fingers with the other player’s fingers”; (10) “I felt like I was competing with the other player”; (11) “I was distracted by the other player’s actions” [for (2), rating 7 = more notes and 1 = less notes; for (6), rating 7 = fast and 1 = slow; for the rest, rating 7 = strongly agree and 1 = strongly disagree]. Error bars represent mean ± SEM; *n* = 20. Download Figure 2-1, TIF file.

10.1523/ENEURO.0133-21.2021.f2-2 Extended Data Figure 2-2 ***A***, ***B***, Time-frequency analysis of the AEP locked to the task-irrelevant beep onsets, presented as the ERSP in ***A*** and the IEC analysis in ***B***. Both ERSP and ITC showed changes in θ activity at 100–350 ms postonset (upper panel). An increase in θ activity (3–7 Hz) was prominent at 150–250 ms postonset (lower panel). ***C***, Topographies of the event-related potential (ERP), bandpass-filtered in the θ range (3–7 Hz), at the indicated time points (ms) from the task-irrelevant beeps showing enhanced potential at the central channels. ***D***, ***E***, The potential, pass-filtered in the θ range (3–7 Hz), at central channels locked to the task-irrelevant beep onsets during the resting (***D***) and playing (***E***) phases. Download Figure 2-2, TIF file.

### Anatomically-defined source estimations

FreeSurfer (Reuter et al., 2012) was used for automatic segmentation and reconstruction of the MRI images. MRI images were used to compute each individualized head model using the boundary element model (BEM) implemented in OpenMEEG withinthe BrainStorm software package (version 3.4) using the default parameters (Gramfort et al., 2010; Tadel et al., 2011). MRI registration with EEG electrode-positions were aligned with each participant’s BEM model, and sources were computed (version 2018) using BrainStorm for each NC epoch in the playing phase. Maps of cortical activity density were obtained across the BEM mesh using the distributed minimum-norm estimate (MNE) method, with constrained dipole orientations and no baseline noise correction. For cortical region-based analysis, brain regions were defined according to the anatomic parcellation of the Destrieux atlas as implemented in FreeSurfer and available in BrainStorm (Destrieux et al., 2010). The time series of source activities from the 15,002 vertices and the averaged activity of the predefined 148 regions of interest (ROIs) were exported for further analysis.

### Power spectrum analysis

The power spectral density (PSD) estimate was calculated using Welch’s overlapped segment averaging estimator as implemented in the MATLAB 2016a signal processing toolbox within the EEGLAB toolbox using default parameters (Welch, 1967; Brunner et al., 2013). The normalized PSD was calculated for each NC epoch then averaged within each trial yielding trial PSD data at each of the 128 channels, the 148 brain region sources, and the 15,002 mesh vertex sources. For each song played, the individual’s mean PSD across the three conditions was calculated. The normalized power was calculated by subtracting the individual’s mean PSD from the PSD at each condition. The normalized power was averaged within the following frequency bands: δ (1–3 Hz), θ (4–7 Hz), α (8−12 Hz), β (13–30 Hz), γ (31–120 Hz), and lower γ (31–50 Hz). We started with exploratory analysis by checking the normalized power grand-averaged across all channels for each frequency band (Extended Data Fig. 3-1). We found significant differences across conditions in the γ (31–120 Hz; one-way repeated measures ANOVA, *F*_(2,57)_ = 5.1445, *p* = 0.0105) band, and showing a trend in the α (8−12 Hz; one-way repeated measures ANOVA, *F*_(2,57)_ = 2.3661, *p* = 0.1075) and β (13–30 Hz; one-way repeated measures ANOVA, *F*_(2,57)_ = 2.0504, *p* = 0.1427) bands. The δ (1–3 Hz; one-way repeated measures ANOVA, *F*_(2,57)_ = 0.5378, *p* = 0. 5884) and θ (4–7 Hz; one-way repeated measures ANOVA, *F*_(2,57)_ = 0.1129, *p* = 0.8936) bands were not significant. For the topographical analysis, the normalized power for the 128-channel data and the permutation statistics with Bonferroni multiple comparison correction were projected to topographical maps using EEGLAB toolbox. As detecting high-γ power (> 50 Hz) using noninvasive EEG might be prone to artifacts (Völker et al., 2018), we only considered the combined β and low-γ (β-γ) band (13–50 Hz) for further analysis. We used one-way repeated measures ANOVA across conditions for determining the significance in each anatomic-source β-γ power effect. We set the significance threshold to *p* < 0.00034 (i.e., 0.05/148 ROIs) to correct for multiple comparisons (Bonferroni-corrected critical value).

### Unsupervised clustering analysis

We clustered the 15,002 mesh vertex sources based on their β-γ power. We used scikit-learn, a Python machine learning library, and implemented the unsupervised agglomerative clustering approach (Abraham et al., 2014). Agglomerative clustering uses a bottom-up hierarchical approach where vertices are progressively linked together into clusters based on their feature similarity. We used three features for clusters which are the grand averaged β-γ normalized power at each of the three conditions. We used the Euclidean distance as a similarity measure and the complete linkage criteria, which minimizes the maximum distance between observations of pairs of clusters. We have tried setting the number of clusters into 3–40 clusters. We selected the minimum number of clusters, seven in our case, that shows trends for flow-related and team-related clusters. When we set the number of clusters to three up to six clusters, the anatomic resolution was not clear. When we set the number of clusters to more than seven clusters, the anatomic resolution was more clear, and we obtained higher significant clusters even after multiple comparison. When we set the number of clusters to seven clusters (cls; Extended Data Fig. 4-2), we detected two cls distributed over the anterior part of the frontal cortex, where the β-γ power was higher in the team only condition than the other conditions (Extended Data Fig. 4-2*C*,*D*; cls 1 and 2). This pattern was significant in cl 2 (one-way repeated measures ANOVA, *F*_(2,57)_ = 3.6125, *p* = 0.033), while cl 1 showed a trend (one-way repeated measures ANOVA, *F*_(2,57)_ = 1.5916, *p* = 0.2125). The suppressed activity in these clusters is specific to the flow experience, regardless of the social context, which is consistent with a neural representation of the automaticity-dimension of flow (Klasen et al., 2012; Ulrich et al., 2014). We also detected two clusters distributed mostly over the middle and inferior frontal cortex and the left occipital cortex (OC), where the β-γ power was lower in the flow only condition than the other conditions (Extended Data Fig. 4-2*C*,*D*; cls 3 and 4). This pattern was significant in cl 4 (one-way repeated measures ANOVA, *F*_(2,57)_ = 7.4841, *p* = 0.0013), while cl three showed a trend (one-way repeated measures ANOVA, *F*_(2,57)_ = 2.4288, *p* = 0.0972). The increased activity in these clusters is specific to team interactions, regardless of the flow state. The remaining clusters were distributed mostly over the temporal, parietal, and occipital cortices, where the β-γ power was higher in the team flow condition than the other conditions (Extended Data Fig. 4-2*C*,*D*; cls 5–7). This pattern was significant in all three cls: cl 5 (one-way repeated measures ANOVA, *F*_(2,57)_ = 11.8753, *p* = 0.000049), cl 6 (one-way repeated measures ANOVA, *F*_(2,57)_ = 9.548, *p* = 0.00027), and cl 7 (one-way repeated measures ANOVA, *F*_(2,57)_ = 6.9256, *p* = 0.002). The increased activity in these clusters was specific to team flow.

### Grouping of ROIs

First, the anatomically-defined ROIs that showed significant β-γ normalized power across conditions, as shown in Extended Data Figure 3-2*A–C*, were grouped as RG7 regardless of their cluster composition. Second, for the remaining anatomic-defined ROI, we calculated the cluster composition as the percentage of the flow-related clusters (cls 1–2), team-related clusters (cls 3–4), and team flow-related clusters (cls 5–7). We checked whether the anatomically-defined ROIs can be spatially subdivided into smaller ROIs with clear tendencies for a certain activity-dependent cluster composition (Extended Data Fig. 4-1*A*). This check was done by calculating a cumulative cluster composition curve to define a threshold for subdividing the ROIs (Extended Data Fig. 4-1*B*). We presented the superior frontal cortex as an example of the subdivided ROIs (Extended Data Fig. 4-1*A*,*B*). Finally, we grouped anatomically-defined ROIs or their subdivisions into six regions (RGs) per hemisphere based on the major activity-dependent cluster composition (Extended Data Fig. 4-1*C*). Therefore, the total number of RGs was 14 RGs (seven RGs per hemisphere). For each of the 14 RGs, the activity-dependent cluster composition is summarized in Extended Data Figure 4-3 and the anatomic composition is summarized in Extended Data Figure 4-4. The time series from all the 15,002 vertices were averaged based on the new 14 RGs and hence reduced into 14 time series for each trial per participant.

### Intrabrain causal interactions analysis

We used the Source Information Flow Toolbox (SIFT) to fit an adaptive multivariate autoregressive (AMVAR) model for the 14 RGs activities for each subject’s trial using the Vieira–Morf algorithm (Delorme et al., 2011). We fitted the NC epoch with a sliding window length of 500 ms and a step size of 25 ms (Wang et al., 2014). Model order was selected by minimizing the Akaike Information criterion. We validated each fitted model using tests included in SIFT for consistency, stability, and whiteness of residuals. To estimate causal interactions, we used three directed model-based linear frequency-domain Granger-causality (GC) measures (Wang et al., 2014). These measures are the normalized partial directed coherence (nPDC; Baccalá and Sameshima, 2001), the direct directed transfer function (dDTF; Korzeniewska et al., 2003), and the Granger–Geweke causality (GGC; Geweke, 1982; Bressler et al., 2007). For each connectivity measure, we averaged across trials for each participant per condition, then averaged across the NC epoch time interval (3 s) and across the β-γ (13–50 Hz) frequency. Finally, to quantify the degree by which an RG sends or receives information, we calculated the ratio of sending (to) divided by receiving (from) for each RG-RG interaction and then average these ratios for each RG per condition per participant (to/from ratio). A two-way repeated measures ANOVA was used as statistical test. To calculate the information senders for RG-RG causal interactions, we used the Log to/from GGC ratio for each RG-RG connection. Top information senders were calculated by setting a threshold with a *p* value of 0.064. The RG-RG connections above this threshold were represented on a circular graph.

### Integrated information analysis

Integrated information (II) was used as a measure of inter-RG bidirectional causal interaction. For every pair of time courses of the RGs activities, within and between participants, we operationalized the “state” of the pair of RGs by discretizing time-samples into binary values. To roughly match the frequency range of 13–50 Hz, we first down-sampled the RGs activities to give timesteps of 12.8, 17.1, 25.6 Hz or 51.2 Hz (that is, a time step of 19.5, 39.1, 58.6, or 78.1 ms). Using the down-sampled RGs activities, we then converted each pair of consecutive time samples to “on” if the RGs activity’s voltage was increasing over two time steps and “off” otherwise. Using the time series of binarized states, we computed the probabilities of each state transitioning into each other state, constructing a transition probability matrix (TPM) which describes the evolution of the pair of RGs activities across time. To ensure accuracy of transition probabilities, we computed these across all trials. As lower time resolutions give fewer observations with which to compute the probabilities, we repeated the down-sampling for each possible “start” (i.e., for each time-sample in the first time-bin) and used all transitions from all shifted-down-sampled time series to build the TPM. We then submitted the TPM to PyPhi (1.2.0; Mayner et al., 2018), which then constructs a minimally reducible version of the TPM, assuming independence of RGs activities, and compares the original TPM to the minimally reducible version to compute II (Oizumi et al., 2014; Mayner et al., 2018). For each actual pair, we calculated the normalized II value by subtracting the absolute value from the average across all conditions for each RG-RG connection. A three-way repeated measures ANOVA (condition × RG1 × RG2) was used as a statistical test for normalized II at each RG-RG connection. The global normalized II was calculated through averaging normalized II values across all possible RG-RG connections. A one-way repeated measures ANOVA was used as a statistical test for global normalized II.

### Phase synchrony analysis

The phase-locking value (PLV), or intersite phase clustering (ISPC), was used as an index of neural synchrony. The distribution of the phase angle differences between sources was generated at each time point (within the NC epoch 3-s window) then averaged over (ISPC-trial; Lachaux et al., 1999; Cohen, 2014). ISPC-trial was calculated at each frequency and then averaged across the frequency band of 13–50 Hz. For each condition, we calculated the ISPC-trial between all sources for the actual pairs or for each of 10 randomly-assigned pairs. For each actual or random pair, we calculated the normalized PLV value by subtracting the PLV value from the average across all conditions for each RG-RG connection. The global normalized PLV was calculated through averaging normalized PLV values across all possible RG-RG connections. A two-way repeated measures ANOVA was used as a statistical test.

### Statistical analysis

All statistics were done using the Statistics and Machine Learning Toolbox within MATLAB 2016a and JASP (Version 0.14.1). We compared non-overlapping dependent correlations, as described in the article (https://garstats.wordpress.com/2017/03/01/comp2dcorr/), using the Robust Correlation Toolbox in MATLAB (http://sourceforge.net/projects/robustcorrtool/) which was validated for Spearman’s correlation (Wilcox, 2016). In this section, we give a parameter justification for each analysis based on the rationale for doing the analysis.

#### Screening process

The screening process is necessary in this study to attain reasonable team flow behavioral response. In the first screening process, we needed to assure that participants who signed up for this study have enough skill to fall into the flow state. In the second screening process, we needed to match participants based on their skill and song preference. We assumed that this screening would maximize the chances of finding pairs of participants who can reach the team flow state.

#### Sample size

The final number of participants was mainly constrained by availability after the screening process. We tried to kept the final number of participations similar to the sample sizes reported in similar publications (Yun et al., 2012). Note, during the main experiment, the data collection process for one male pair of participants was interrupted because of a technical error, and the collected data were excluded from data analysis.

#### Trial numbers

We limited the number of trials to six per condition to avoid fatigue which might have compromised the possibility of falling into the flow state in later trials. For one pair, we could only collect five trials per condition because of time constraints. For another pair, one of the trials contained excessive noise, and hence, we excluded this trial and all corresponding trials in the other conditions.

#### Units of analysis

Unless otherwise described, the unit of analysis is participant, i.e., *n* = 15. For the five participants invited twice, we averaged the results from the two experiments giving one data point. In some analyses, the unit of analysis was participation, i.e., *n* = 20. For the performance analysis, the unit of analysis was the final score for the pair, i.e., *n* = 10. Data collection was not performed blind to the conditions of the experiment. Experimental blinding was not possible because of the overt and obvious nature of the experimental setup for each manipulation. Data in all conditions were subjected to identical analysis algorithms.

### Data availability

Analysis codes used in the preparation of this article are available at https://osf.io/3b4hp.

## Results

### Behavioral paradigm for team flow

We designed a behavioral paradigm to assess team flow, in which a pair of participants played a popular music rhythm game. The game’s task required responses by tapping a touch screen when animated visual cues reached a designated area and delivered instantaneous positive feedback. The game created the impression of playing a musical instrument, which increases the likelihood of entering a flow state. Each pair of participants played as a team by splitting the tapping area and sharing in task completion with the common goal of obtaining the best score for the team. We simultaneously recorded their brain activities using electroencephalogram (EEG; Fig. 1*A*; Extended Data Fig. 1-1; Movie 1). Participants were screened to select prosocial highly-skilled participants in this game and were matched according to their skill level and song preference (for more details, see Materials and Methods).

In the primary experimental condition, the team flow condition, teams played the unmodified songs in an open interpersonal setting to maximize the team flow experience (Fig. 1*B*, left panel). To fulfill the team characteristics: (1) common purpose: we instructed each pair of participants (team) to get the highest score for the team; (2) complimentary skills: we matched participants based on skill and song preference; (3) clear performance goals: we provided the performance feedback at the end of each trial; (4) keep a strong commitment: we allowed for the visibility of teammate’s instant feedback; and (5) mutual accountability: we explained that a decrease in performance from any teammate would affect the total score. We designed two control conditions to manipulate either the flow or the social states. To disrupt the flow state, we manipulated the team only condition by modulating the intrinsic reward/enjoyment dimension for flow by scrambling the game’s music. This procedure then interrupted the sense of immersion and the continuity of the game (Fig. 1*B*, middle panel). To disrupt the social state in the flow only condition, we used an occlusion board between the two participants that occluded the partner’s positive feedback and bodies while leaving all of the cues visible to both players (Fig. 1*B*, right panel; Extended Data Fig. 1-1*A*). We designed the manipulations to disrupt one component of team flow per condition to ensure that any discovered NC for team flow did not arise from only one of these components.

To control for stimuli-related neural activities, we kept all stimuli constant across conditions by asking the teams to play the same song at the three conditions (Fig. 1*C*). For each song, the visual stimulus (cue sequence), the total auditory stimulus presented, task difficulty, and the sequence of the task-irrelevant beeps were kept constant across conditions (Table 1). Then we normalized the neural signals per song. The only remaining variables across conditions were the song’s pleasance and the visibility of the partner’s positive feedback. There were no differences in the participants’ performances across conditions (repeated measures one-way repeated measures ANOVA, *F*_(2,27)_ = 0.02437, *p* = 0.976), which ensure no differences in gross motor responses.

### Subjective assessment of team flow

To validate our manipulations, participants performed psychometric ratings after each trial (Fig. 1*D*; Extended Data Fig. 1-1*B*). To assess the dimensions of the flow state, we presented the participants with the following psychometric ratings: (1) “I had the necessary skill to play this trial successfully”; (2) “I will enjoy this trial more if it has less/more notes”; (3) “I felt in control while playing this trial”; (4) “I made correct movements automatically without thinking”; (5) “I love the feeling of this trial and want to play it again”; and (6) “How time flies during this trial.” To assess positive social interaction for teams, we presented the following: (7) “I was aware of the other player’s actions”; (8) “I felt like I was playing with the other person as a team”; and (9) “I was coordinating my fingers with the other player’s fingers” (Extended Data Fig. 2-1). Responses were collected on a seven-point Likert scale and averaged into a flow index by averaging responses across (1) to (6), a team index by averaging across (7) to (9), and a team flow index by averaging across (1) to (9). As expected, the flow index decreased significantly in the team only condition than the other two conditions (Friedman test, non-parametric repeated measures ANOVA, χ^2^ = 20.133, *p* < 0.001, *n* = 15; Fig. 2*A*). The team index decreased significantly in the flow only condition than the other two conditions (Friedman test, χ^2^ = 20.373, *p* < 0.001, *n* = 15; Fig. 2*B*). The team flow index was significantly higher in the team flow condition more than the other two conditions (Friedman test, χ^2^ = 22.933, *p* < 0.001, *n* = 15; Fig. 2*C*). The results of the psychometric assessment confirmed effectiveness of our manipulations to achieve the desired subjective experience for each condition.

### Objective assessment for the depth the flow state

To provide objective evidence for the flow state, we developed a novel neurophysiological measure of flow. We used the intense task-related attention and the reduced sense of external awareness dimensions of flow (Nakamura and Csikszentmihalyi, 2002), and the well-known effect of selective attention on the AEP (Picton and Hillyard, 1974). During each trial, we presented task-irrelevant beeps to the participants (Fig. 1*D*; Extended Data Fig. 1-1*B*). The more the participants were immersed in the game, the weaker the strength of the AEP in response to the task-irrelevant beeps. Thus, this AEP constitutes an objective measure for flow (Fig. 2*D*,*E*; Extended Data Fig. 2-2). The mean AEP response was significantly higher in the team only (mean = 0.29, 95% confidence interval (CI) [0.22, 0.35]) condition more than the other two conditions (team flow mean: 0.19, 95%CI [0.13, 0.25]; flow only mean: 0.17, 95%CI [0.11, 0.24]; one-way repeated measures ANOVA, *F*_(2,42)_ = 6.149, *p* = 0.006, η^2^ = 0.305; Fig. 2*D*). The higher AEP in the team only condition indicated that the participants where not fully engaged, and hence their brains responded more to the task-irrelevant beep sound. Notably, the AEP was negatively correlated with the flow index in the team flow condition (Spearman’s Rho = −0.48 [−0.76, −0.03], *p *=* *0.03), while it was only weakly (Spearman’s Rho = −0.29 [−0.66, 0.14], *p *=* *0.22) or not correlated (Spearman’s Rho = 0.11 [−0.35, 0.55], *p *=* *0.64) with the flow index in the flow only and the team only condition, respectively (Fig. 2*E*). The negative correlation of AEP with the flow index was significantly stronger in the team flow condition than the team only condition (Spearman’s Rho difference = 0.59 [−1.05, −0.05], *p* = 0.04). These results indicate that the experimental manipulations did produce a deeper flow state in the team flow and flow only conditions than the team only condition.

### β-γ Power at the middle temporal cortex (MTC) as a neural signature for team flow

**Figure 3.**
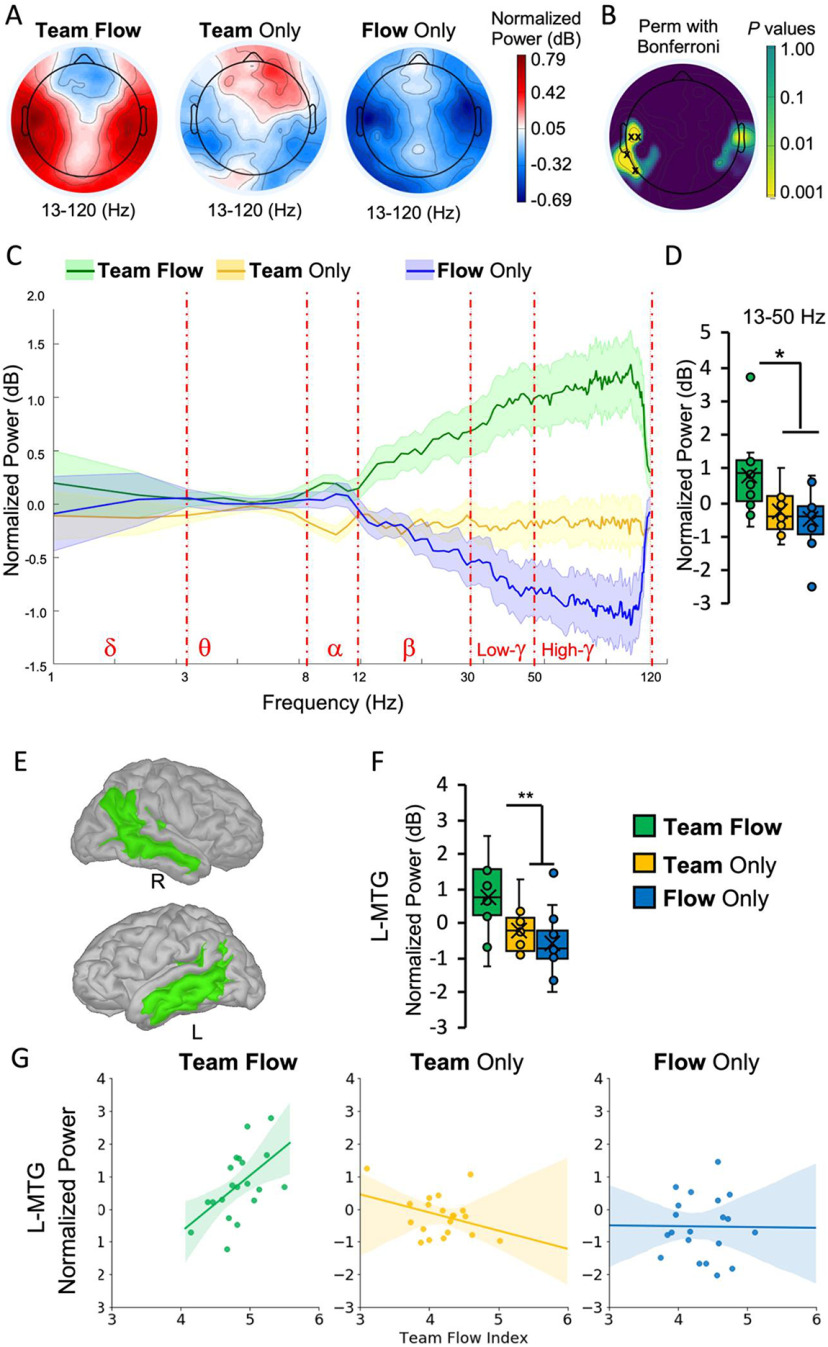
Higher β-γ power at the L-MTC revealed as a unique neural signature for team flow. ***A***, The topographies of the β and γ frequencies (13–120 Hz) computed as the average over normalized power. ***B***, Permutation statistical significance across conditions with Bonferroni multiple comparison corrections. The black crosses indicate channels with *p* < 0.05. ***C***, The normalized power spectral analysis averaged from the four channels in the left temporal area identified in ***B***. Shaded area represent mean ± SEM; *n* = 20. Extended Data Figure 3-1 shows the power difference spectral analysis grand averaged across all the 128 channels. ***D***, Averaged normalized power for the β-γ (13–50 Hz) frequency band showing power enhancement in the team flow condition. One-way repeated measures ANOVA with Bonferroni *post hoc* test; *p < 0.05. Error bars represent mean ± SEM; *n* = 15. ***E***, The brain regions (highlighted in green), as defined by the Destrieux atlas and showing significant β-γ normalized power difference across conditions. Extended Data Figure 3-2 shows the average normalized β-γ power for each significant region. ***F***, The average normalized β-γ power at the L-MTG. One-way repeated measures ANOVA with Bonferroni *post hoc* test; **p < 0.01. Error bars represent mean ± SEM; *n* = 15. ***G***, Condition-specific Spearman’s correlations between β-γ power and team flow index at L-MTG as a representative region. Positive correlation was found in the team flow condition (Spearman’s Rho = 0.56, *p* = 0.006), but not in the team only condition (Spearman’s Rho = −0.19, *p* = 0.43) or in the flow only condition (Spearman’s Rho = −0.02, *p* = 0.95). The lines indicate the regression lines. Shaded areas indicate a 95% confidence interval; *n* = 20.

To detect specific NCs for team flow, we used power spectral analysis at various domains (Extended Data Fig. 1-1*B*). In the frequency domain, we started with exploratory analysis by checking the normalized power grand-averaged across all channels for each frequency band. We found significant differences across conditions in the α (8–12 Hz), β (13–30 Hz), and γ (31–120 Hz) bands (for details, see Materials and Methods, Power spectrum analysis; Extended Data Fig. 3-1). At the topographical domain level, α power analysis did not show specific surface channels significantly different across conditions (data not shown). Topographical β-power and γ-power analysis showed four channels at the left temporal area with significantly higher β and γ power in the team flow condition, more than the other two conditions (Fig. 3*A*,*B*). The power spectral analysis, averaged from these four channels, showed a clear higher normalized β-power and γ-power in the team flow condition than the other conditions (Fig. 3*C*). As there are some limitations in the capability of EEG to accurately detect high-γ (> 0 Hz) power, we used the combined β and low-γ (β-γ) band (13–50 Hz) for further analysis. The β-γ band showed significantly higher normalized power in the team flow condition, more than the other two conditions (Fig. 3*D*, one-way repeated measures ANOVA, *F*_(2,42)_ = 6.335, *p* = 0.005, η^2^ = 0.312; team flow mean: 0.77, 95%CI [0.32, 1.23]; team only mean: –0.27, 95%CI [−0.72, 0.19]; flow only mean: –0.51, 95%CI [−0.96, −0.06]).

10.1523/ENEURO.0133-21.2021.f3-1 Extended Data Figure 3-1 ***A***, The power difference spectral analysis for the three conditions grand averaged for all the 128 channels. ***B***, Averaged individual power difference for the α (8–12 Hz), the β (13–30 Hz), and γ (31–120 Hz) frequency bands. One-way repeated measures ANOVA with Bonferroni *post hoc* test; *p < 0.05. Error bars represent mean ± SEM; *n* = 20. Download Figure 3-1, TIF file.

10.1523/ENEURO.0133-21.2021.f3-2 Extended Data Figure 3-2 Localization of the higher β-γ power during team flow. ***A***, The brain regions (highlighted in green), as defined by the Destrieux atlas and showing significant β-γ normalized power difference across conditions. ***B***, The average normalized β-γ power at the significant ROIs. Bonferroni-corrected critical value one-way ANOVA with Bonferroni *post hoc* test. ***C***, ***D***, Unsupervised hierarchical vertices clustering based on β-γ power difference between conditions. ***C***, Clustered-vertices projected to a standard brain to visualize cluster localization. The black lines indicate the boundaries of the ROIs shown in ***B***. ***D***, The cluster-averaged normalized power of the β-γ power at each cluster. One-way repeated measures ANOVA with Tukey–Kramer’s *post hoc* test. Flow-related (cl 1–1–2), social-related (cls 3–4), or team flow-related (cls 5–7) clusters are indicated in the same color scheme as in ***D***; *p < 0.05, **p < 0.01, #*p* = 0.077. Error bars represent mean ± SEM; *n* = 20. B, bottom view; R, right; L, left; AOS, anterior occipital sulcus; PLF, posterior lateral fissure; ITS, inferior temporal sulcus; MTG, middle temporal gyrus; STS, superior temporal sulcus; PT, superior plannar-temporal gyrus; TPJ, temporal parietal junction, LTS, lateral temporal sulcus; ITG, inferior temporal gyrus; CLS, collateral and lingual sulcus. Download Figure 3-2, TIF file.

At the anatomic-source domain level, we performed a cortical source localization method, using co-registration with the individual’s structural MRI. The brain was segmented into 148 ROIs based on the Destrieux brain atlas (Destrieux et al., 2010). The anatomic-source β-γ power analysis, after multiple comparison correction, showed 16 ROIs in the left and right temporal areas with a significantly higher β-γ power in the team flow condition compared with the other two conditions (Extended Data Fig. 3-2*A*,*B*). As a representative example, the normalized β-γ power for the left middle temporal gyrus (L-MTG) is shown in Figure 3*F* (one-way repeated measures ANOVA, *F*_(2,42)_ = 6.744, *p* = 0.004, η^2^ = 0.325; team flow mean: 0.76, 95%CI [0.32, 1.2]; team only mean: –0.2, 95%CI [−0.63, 0.24]; flow only mean: –0.56, 95%CI [−1.0, −0.12]). Also, the β-γ power of these brain regions showed higher correlation tendencies with the team flow index only in the team flow condition. The L-MTG showed the highest β-γ power correlation with the team flow index in the team flow condition (Spearman’s Rho = 0.59 [0.21, 0.84], *p* = 0.006) but not in the team only (Spearman’s Rho = −0.19 [−0.67, 0.34], *p* = 0.43) or the flow only (Spearman’s Rho = −0.02 [−0.46, 0.42], *p *=* *0.95) conditions (Fig. 3*G*). The positive correlation of the β-γ power with the team flow index was significantly higher in the team flow condition than the team only condition (Spearman’s Rho difference = 0.78 [−0.01 1.40], *p* = 0.05) and the flow only condition (Spearman’s Rho difference = 0.61 [0.00, 1.16], *p* = 0.05).

We note that some ROIs showed a trend unique to the team only or the flow only conditions, but they did not survive after the multiple comparison correction. Since the anatomic-source localization averages source vertices based on a predefined parcellations method, we developed a method to give more weight to the distribution of activity rather than anatomy. We used unsupervised machine learning to cluster (cl) the source vertices based on their similarity in the β-γ power pattern (Extended Data Fig. 3-2*C*,*D*). Using the unsupervised clustering analysis, we detected cls specific to team flow, team only and flow only conditions (for details, see Materials and Methods, Unsupervised clustering analysis). These results indicate that even during team flow, the brain shows neural activities related to each isolated experience: the flow and the social states.

The results from the power spectral analyses at every tested domain provided the first neural evidence that the team flow experience is a qualitatively different brain state distinguishable from the flow or social states. In other words, the team flow state does not result from a simple combination of the flow and the social states, but it has its own neural signature, which we posit accounts for the superiority in the subjective experience. Next, we checked for possible unique interactions between these brain regions during team flow. Before performing further analyses, we grouped the 148 ROIs into 14 brain regions, seven per hemisphere, using a combination of the standard anatomic definition and the functional activity revealed through the cluster analysis (Extended Data Figs. 3-2*C*,*D*, 4-1, 4-3, and 4-4). These 14 brain regions (RGs) are: the PFC (RG1), the ACC (RG2), the inferior frontal cortex (IFC, RG3), the superior temporal cortex (STC; RG4), the central and parietal cortex (CPC; RG5), the OC (RG6), and the MTC (RG7). The MTC included all the ROIs that showed a significant effect on team flow (Extended Data Fig. 3-2*B*), regardless of the cluster composition.

### The left MTC (L-MTC) receives and integrates information from brain areas encoding flow or social states

**Figure 4.**
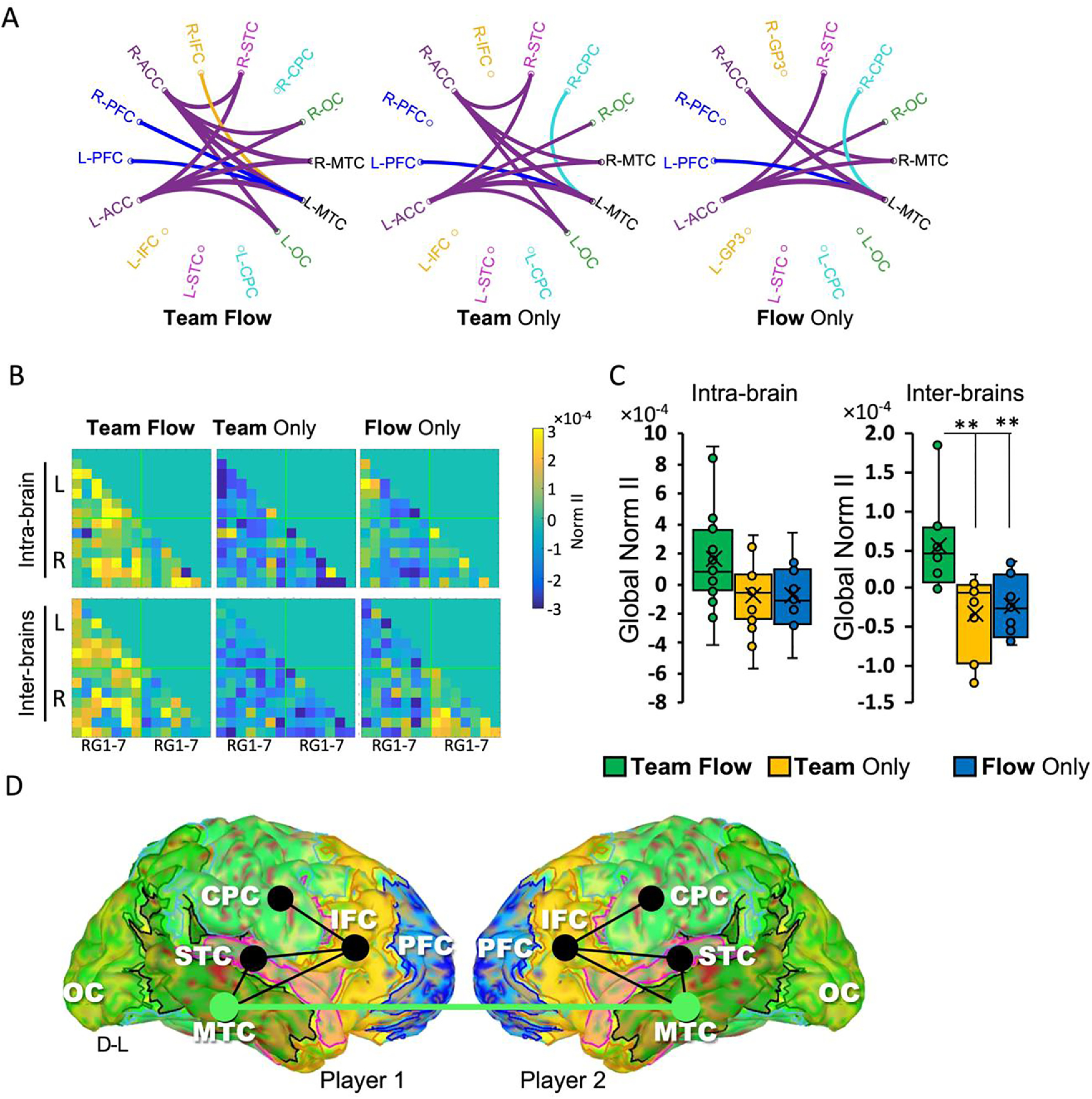
Causality and II analyses during team flow. ***A***, Causality analysis showing the top information senders among all RG-RG causal interactions. For each RG-RG connection, the line color matches the color of the RG name which sends the information. Notably, only in the team flow condition, L-MTC receives information from R-PFC and R-IFC. Extended Data Figures 4-1, 4-3, 4-4 show the method for grouping of ROIs. Extended Data Figure 4-2 shows detailed causality analysis. ***B***, The mean normalized II value (Norm II) connectivity matrix for the brain regions (RG1–RG7). Normalized II is calculated by subtracting the mean per condition from the average II across conditions for each RG-RG connection across conditions. ***C***, The mean global Norm II averaged across all RG-RG connections showing significantly higher interbrain (left panel) and intrabrain (right panel) mean during team flow condition. One-way repeated measures ANOVA with Bonferroni *post hoc* test; **p < 0.01. Error bars represent mean ± SEM; *n* = 15. ***D***, RG-RG connections that shows significant (*p* < 0.05) Norm II in the team flow condition compared with other conditions. Three-way repeated measures ANOVA with Bonferroni *post hoc* test. Black lines indicate intrabrain and green line indicates interbrain RG-RG connections. D-L, dorsal-left.

We tested whether the neural signature of team flow detected in the MTC upstream or downstream in information processing. We analyzed the causal information interactions across all the brain regions (RGs), using three frequency-domain GC measures: the GGC, the dDTF, and the nPDC (Wang et al., 2014). In all GC measures, the causal interaction matrix showed that MTC receives information (from) more than sending information to (to) other RGs (Extended Data Fig. 4-2*A*). Also, we quantified the global to/from ratio for each RG per condition. In all GC measure, global to/from ratio for the L-MTC was significantly less than any other RG except for the right MTC (R-MTC; Extended Data Fig. 4-2*B*; for GGC, two-way repeated measures ANOVA, *F*_(26,494)_ = 2.9768, *p* = 0). Hence, the detected β-γ power in L-MTC is a downstream in information processing during the team flow experience. We then checked the most important upstream brain regions that sent information to L-MTC. For each RG-RG causal interaction, we applied a global threshold to leave only the top (∼10%) information senders (Fig. 4*A*). Only in the team flow condition, the top information senders to L-MTC include the contralateral R-PFC and R-IFC. The team only and flow only conditions showed a similar causality pattern, yet different from the team flow condition, in which the top information senders to L-MTC include the contralateral R-CPC. Interestingly, the differences between the conditions were observed in the interhemispheric connectivity rather than the intrahemispheric one.

10.1523/ENEURO.0133-21.2021.f4-1 Extended Data Figure 4-1 Activity-dependent anatomically-defined grouping of ROIs (RGs). ***A***, A medial view of the left superior frontal cortex (area inside the red boundary/transparent contour) before (left panel) and after (right panel) subdivision. ***B***, The cumulative cluster composition curve for the left superior frontal cortex. The subdivision thresholds are shown as two vertical black lines subdividing this ROI into three subdivisions: flow-related subdivision (cls 1–2), team-related subdivision (cls 3–4), and team flow-related subdivision (cls 5–7). ***C***, Transparent contours showing the brain regions which are also summarized in Extended Data Table 4-2. B, bottom; D, dorsal; L, left; R, right; T, top; V, ventral. Download Figure 4-1, TIF file.

10.1523/ENEURO.0133-21.2021.f4-2 Extended Data Figure 4-2 Information causality analysis showing the MTC receives information from other brain regions. ***A***, The mean causal interaction matrix for the brain regions (RGs). “To” indicates sending information; “from” indicates receiving information. The GGC (top), dDTF (middle), and the dDTF (bottom). L, left hemisphere; R, right hemisphere. ***B***, The mean causal to/from ratio for GGC (top), dDTF (middle), and nPDC(bottom). In all GC measure, L-MTC (L-RG7) is a significant information receiver. Two-way repeated measures ANOVA with Tukey’s *post hoc* test; *p < 0.05, ***p < 0.001, ****p < 0.0001. Dashed line indicates *p* > 0.05. Error bars represent mean ± SEM; *n* = 20. Download Figure 4-2, TIF file.

10.1523/ENEURO.0133-21.2021.f4-3 Extended Data Figure 4-3 Cluster composition (percentage) of the activity-dependent anatomically-defined groups (RGs). Download Figure 4-3, DOCX file.

10.1523/ENEURO.0133-21.2021.f4-4 Extended Data Figure 4-4 Anatomical composition of the activity-dependent anatomically-defined groups (RGs). Download Figure 4-4, DOCX file.

The results above indicate that the β-γ power detected in the L-MTC during team flow might arise from information processing that happened earlier in time, and the sources of this information include brain areas that encodes flow state (namely, PFC) and social state (namely, IFC). We suspected that a downstream brain region plays a role in integrating information from the brain regions encoding each isolated experience. To test this hypothesis, we used the II theory (Tononi, 2004; Oizumi et al., 2014). We calculated the normalized II value (Norm II) as a metric for the II. In both intrabrain and interbrain calculations, there was a general tendency for Norm II to be higher in the team flow condition than the other conditions (Fig. 4*B*). When we averaged the Norm II across all the RG-RG connections (global Norm II) from the left hemisphere, the team flow condition showed significant higher interbrain global Norm II than other conditions (one-way repeated measures ANOVA, *F*_(2,42)_ = 9.310, *p* < 0.001, η^2^ = 0.399; team flow mean: 0.56, 95%CI [0.3, 0.83]; team only mean: –0.33, 95%CI [–0.6, –0.06]; flow only mean: –0.23, 95%CI [–0.5, 0.04]), while showing a similar trend at the intrabrain level (one-way repeated measures ANOVA, *F*_(2,42)_ = 2.496, *p* = 0.101, η^2^ = 0.151; Fig. 4*C*).

Next, we checked for the specific RG-RG connections that showed a significant Nom II at the team flow condition compared with other conditions using the three-way repeated measures ANOVA with Bonferroni’s multiple comparison correction (condition × RG × RG interaction for intrabrain: *F*_(26,10133)_ = 4.7622, *p* = 0, and for interbrain: *F*_(26,10959)_ = 3.676, *p* = 0). Among all RG-RG connections, we detected significant connections only at the left hemisphere. These connections formed an intrabrain L-IFC-STC-CPC-MTC subnetwork and an interbrain L-MTC-to-L-MTC link that showed significantly higher Norm II in the team flow than the other conditions (Fig. 4*D*). These results indicate that during team flow, the team members exhibited higher information integration not only within each player’s brain, but also between their brains. More specifically, L-MTC was the only brain region that showed significantly higher interbrain II during team flow. These results indicates that L-MTC plays a critical function in information integration during the team flow state.

### Team flow is associated with higher interbrain neural synchrony

**Figure 5.**
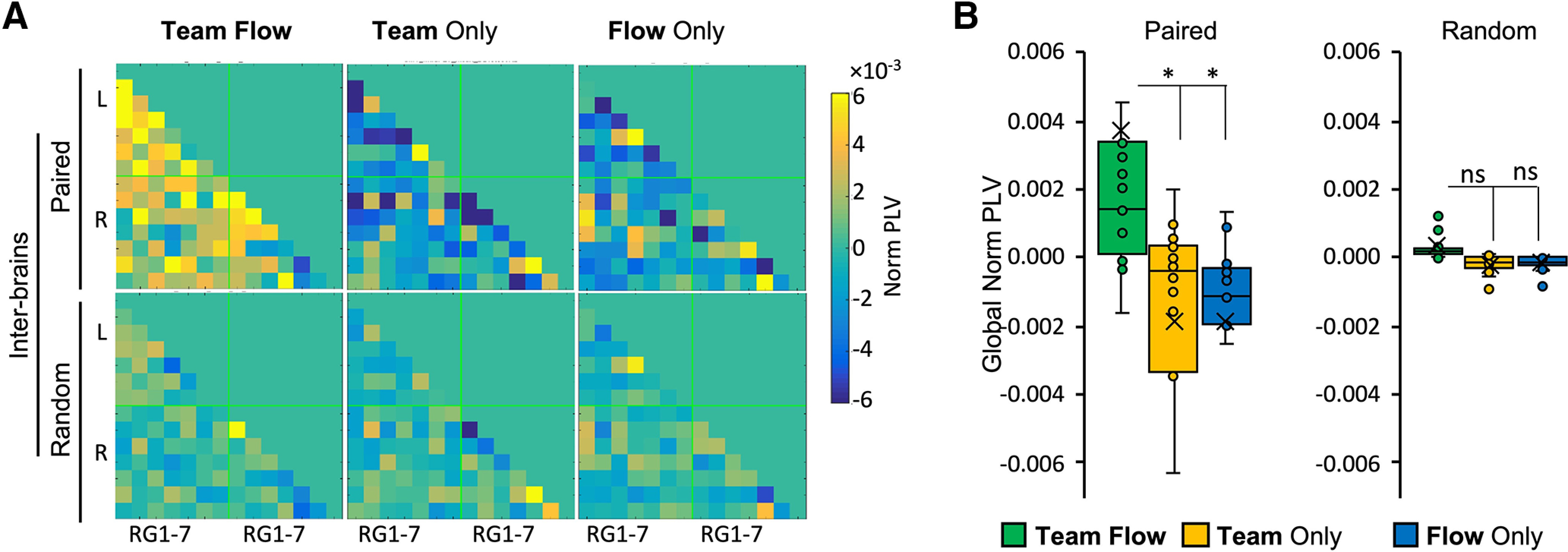
PLVs show enhanced interbrain synchrony during team flow. ***A***, The mean PLV connectivity matrix for the brain regions (RG1–RG7). Normalized PLV is calculated by subtracting the mean per condition from the average PLV across conditions for each RG-RG connection across conditions. “Paired” indicates the actual experimental pair, “random” indicates randomly selected pairs. ***B***, The mean global normalized PLV averaged across all RG-RG connections showing significantly higher interbrains during the team flow condition. Two-way repeated measures ANOVA for interbrain comparison with Bonferroni *post hoc* test; *p < 0.05. Error bars represent mean ± SEM; *n* = 15. ns, not significant.

Enhanced interbrain II might concur with enhanced neural synchrony between the team’s brain regions. To test for this hypothesis, we calculated the interbrains normalized PLV (Norm PLV) across all the RG-RG connections for each condition (Fig. 5*A*). The results showed a general tendency for Norm PLV to be higher in the team flow condition than other conditions. The interbrain Norm PLV calculated using a randomly shuffled pairs did not show any difference across conditions (Fig. 5*A*). To quantify this effect, we averaged the Norm PLV for all RG-RG connections (global Norm PLV) in the left hemisphere. The team flow showed a significantly higher global Norm PLV than other conditions only in the actual paired participants but not in randomized pairs (Fig. 5*B*; two-way repeated measures ANOVA, condition × randomness interaction *F*_(2,84)_ = 3.317, *p* = 0.05, η^2^ = 0.092; condition effect *p* = 0.015, η^2^ = 0.135, team flow mean: 0.002, 95%CI [0.0007, 0.003]; team only mean: –0.001, 95%CI [–0.002, 0.0003]; flow only mean: –0.001, 95%CI [–0.002, 0.0003]). Collectively, these results indicate that during team flow, the team members exhibited higher integration and neural synchrony between their brains. This enhancement in information integration and neural synchrony is consistent with the phenomenological experience during team flow, and it might be the neurocognitive basis for the superior subjective experience of team flow.

## Discussion

In summary, we established a new objective neural measure of flow, consistent with subjective reports. We identified unique NCs of team flow in the β-γ band at the L-MTC. We showed that L-MTC is downstream in information processing and plays a role in information integration in team flow. Finally, team flow is characterized by higher information integration and neural synchrony. The data from this report present a proof of concept that team flow is indeed a distinct brain state and suggests a neurocognitive mechanism of team flow.

### Measuring the depth of the flow experience with parametric tools

To date, studies identified the flow state using only subjective psychometric ratings, which we have reproduced in this report (Fig. 2*A–C*; Extended Data Fig. 2-1; Klasen et al., 2012; Ulrich et al., 2014, 2016a,b). Although psychometric ratings provided some evidence that the participants reached some aspects of the flow experience, they did not indicate the depth of the flow state (Harris et al., 2017). Using the task-irrelevant AEP, we confirmed that our task did attain enough depth of flow experience to alter a key flow dimension: attenuated consciousness to an external stimulus. We note that this measure is not specific to team flow, as it can also measure the general flow state. The task-irrelevant AEP correlated with the Flow Index only in the team flow condition and showed only a trend in the flow only condition. This result suggests that the observed level of flow state in the team flow condition can be stronger than the flow only condition. The flow experience is hypothesized to be either an abrupt discrete zone or a gradual continuum (Ulrich et al., 2014; Harris et al., 2017). Solving this ambiguity will significantly advance our mechanistic understanding of how the flow experience develops and functions. Our newly developed method for measuring the flow depth will be a useful parametric tool for further studies in this area.

### NC of team flow

The most prominent NC for team flow state that we identified was the higher β-γ power in the TMC, as shown in Figure 3. β And γ oscillations are involved in several cognitive functions, including attention, memory, and awareness, with evidence of abnormalities in brain disorders (Uhlhaas and Singer, 2006). In general, these functions are consistent with higher team interactions and enhancing many flow dimensions. Moreover, our data agree with other reported neural activities that study flow state or social interaction using different tasks. For example, in the PFC, the β-γ power was lower in the flow conditions compared with the non-flow conditions (Extended Data Fig. 3-2*C*,*D*). These data agree with the reduced activities in the medial PFC in an arithmetic task (Ulrich et al., 2014, 2016b). In the IFG, the β-γ power was higher in the team conditions compared with the non-team conditions (Extended Data Fig. 3-2*C*,*D*). These data agree with the involvement of the IFG in social interaction in a plethora of different tasks (Kennedy and Adolphs, 2012; Stanley and Adolphs, 2013).

Our data also show that L-MTC falls downstream of other brain areas and receives information from brain areas encoding flow and social states. Also, L-MTC was the only significant region showing higher II during team flow at both the intrabrain and interbrain levels. Previous reports also suggest an integration function for the temporal cortex in different contexts. For example, the middle and inferior temporal gyrus have been reported to play a role in cognitive-affective integration in schizophrenia (Tseng et al., 2015). Thus, our results and past reports fall in line to suggest a neural model during team flow where the L-MTG is involved in integrating the flow and social information to serve the team flow experience.

### Team flow as an independent interbrain state

Recent social neuroscience studies measured the interactions between the brains of team members using interbrain synchronization (e.g., phase synchrony). Group activities can enhance this synchrony during intense social states, body or speech coordination, music production, dancing, student-teacher interactions in classrooms, touch-mediated pain reduction, creativity in cooperative tasks, and even in socially interacting bats (Lindenberger et al., 2009; Dumas et al., 2010; Sanger et al., 2012; Yun et al., 2012; Kawasaki et al., 2013; Dikker et al., 2017; Goldstein et al., 2018; Poikonen et al., 2018; Lu et al., 2019; Zhang and Yartsev, 2019). Importantly, teams exhibit higher interbrain synchrony compared with solo performers (Reinero et al., 2021). We posit that interbrain synchrony can be a metric for more effective group interactions. Similarly, II, which measures the amount of information generated by the system compared with its individual parts, is another metric of group interaction (Oizumi et al., 2014). The interbrain II may predict effective group interaction and complexity, and may serve as a measure of collective intelligence (Engel and Malone, 2018). Based on both metrics, our data indicate that team flow creates a hyper-cognitive state between the team members, as reflected in significantly higher interbrain information integration and neural synchrony during team flow (Figs. 4 and 5). Based on our findings, we cannot conclude that the high value of II correlates with a modified form of consciousness (Koch and Tononi, 2013), for instance, “team consciousness.” Its consistency with neural synchrony (PLV) raises intriguing and empirical questions related to interbrain synchrony and information integration and altered state of consciousness.

### Limitations

In this study, we used strict selection criteria to ensure that participants experienced team flow. During pilot studies, we observed the expression of anti-social behavior by some participants during the game. As these participants were likely on the population extremes and preferred solo rather than team play, we excluded such participants in our team-based experiment. We reasoned that excluding anti-social participants removes noise without biasing the data. A limitation in the strict selection criteria is the generality of the conclusions to the general population. Future studies should test both the social and anti-social participants and compare their neural data during team flow. Another related limitation is the critical question: whether the NCs of team flow are general regardless of the task or only apply to the task, we employed here, i.e., the music rhythm game. Based on the agreement with previous reports as mentioned above, the NCs seem to be not constrained to a specific task but instead support functions related to team flow. However, only future studies using a variety of tasks can confirm this hypothesis. Another limitation in this study is that we did not detect a difference in the participants’ performance across conditions, although the psychometric ratings showed significant changes in the flow index. The decreased performance likely did not occur, as the two flow conditions flanked the team only condition, with one trial duration being short. We think in the future, it is better to implement a block design so the participants can experience an accumulation of experience over time which will have a substantial impact on performance.

## Synthesis

Reviewing Editor: Alexander Soutschek, Ludwig-Maximilians-Universität München Decisions are customarily a result of the Reviewing Editor and the peer reviewers coming together and discussing their recommendations until a consensus is reached. When revisions are invited, a fact-based synthesis statement explaining their decision and outlining what is needed to prepare a revision will be listed below. The following reviewer(s) agreed to reveal their identity: Mari Tervaniemi, Guillaume Dumas.

The reviewers agree that the authors successfully addressed all of their previous concerns.
