# Supplementary figures and images for: Team Flow Is a Unique Brain State Associated with Enhanced Information Integration and Interbrain Synchrony
Source: eNeuro. 2021 Oct 12;8(5):ENEURO.0133-21.2021. doi: 10.1523/ENEURO.0133-21.2021 (PMC8513532; doi:10.1523/ENEURO.0133-21.2021)

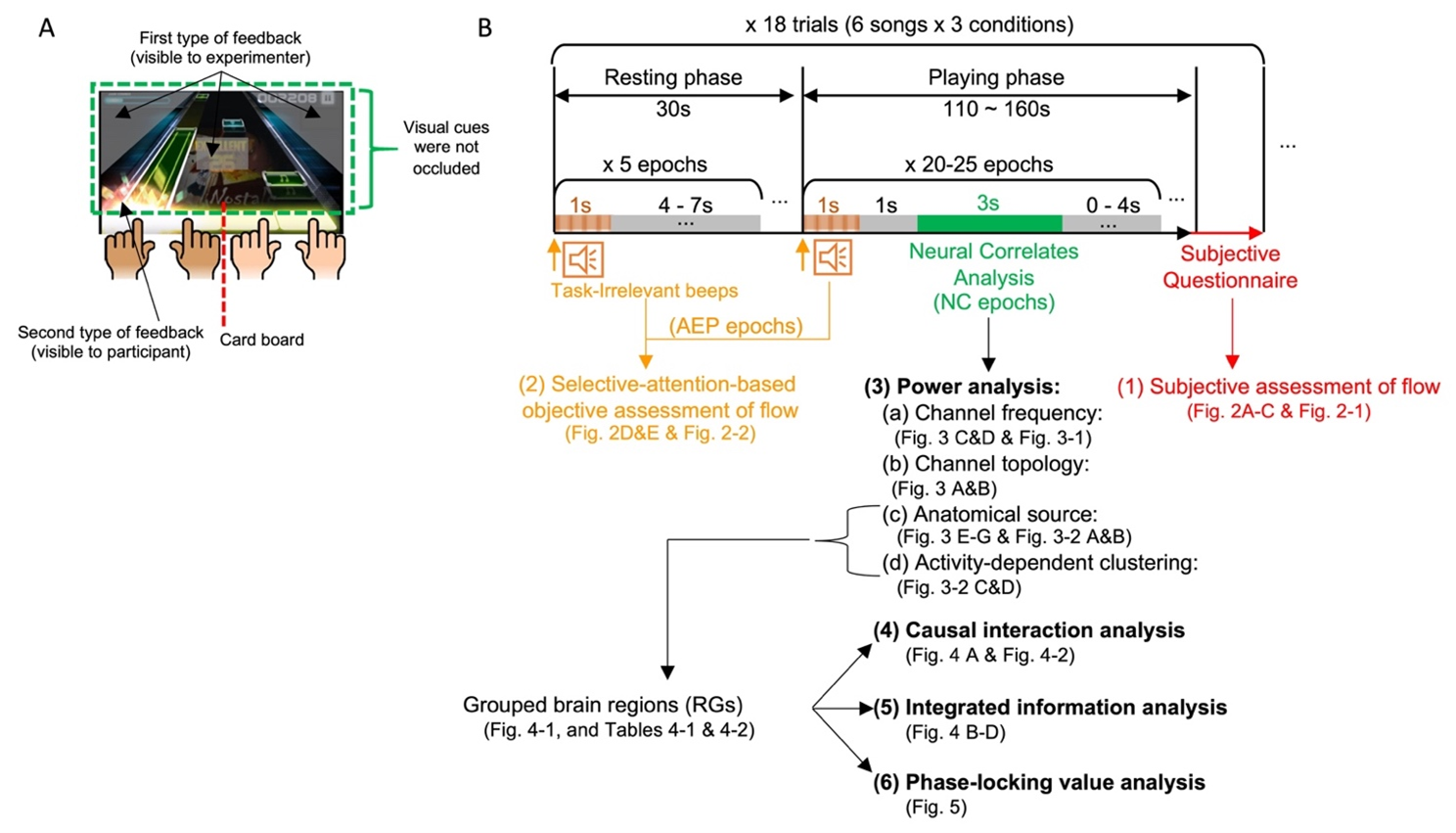

Supplement: Extended Data Figure 1-1 — Experimental design. A, A screenshot of the game showing feedback and position of the card board. The first type of feedback was invisible to participants. The second type of feedback was visible to participants in the Inter-SyncA and Inter-ScrA conditions. The cardboard (red dashed line) occluded the tapping area, the second type of feedback, and the whole bodies of participants including fingers. The cardboard kept the visual cues visible to participants (green dashed rectangle). B, Trial and analyses details: participants were sitting still while listening to a background music during the resting phase. The electroencephalogram was epoched for the AEP analysis of the task-irrelevant beep sound (AEP epochs; orange) and for the NCs analysis (NC epochs; green). All NC analyses, with the corresponding figure or table, are summarized in steps 3–6. Download Figure 1-1, TIF file. [file enu-eN-NWR-0133-21-s04.tif]

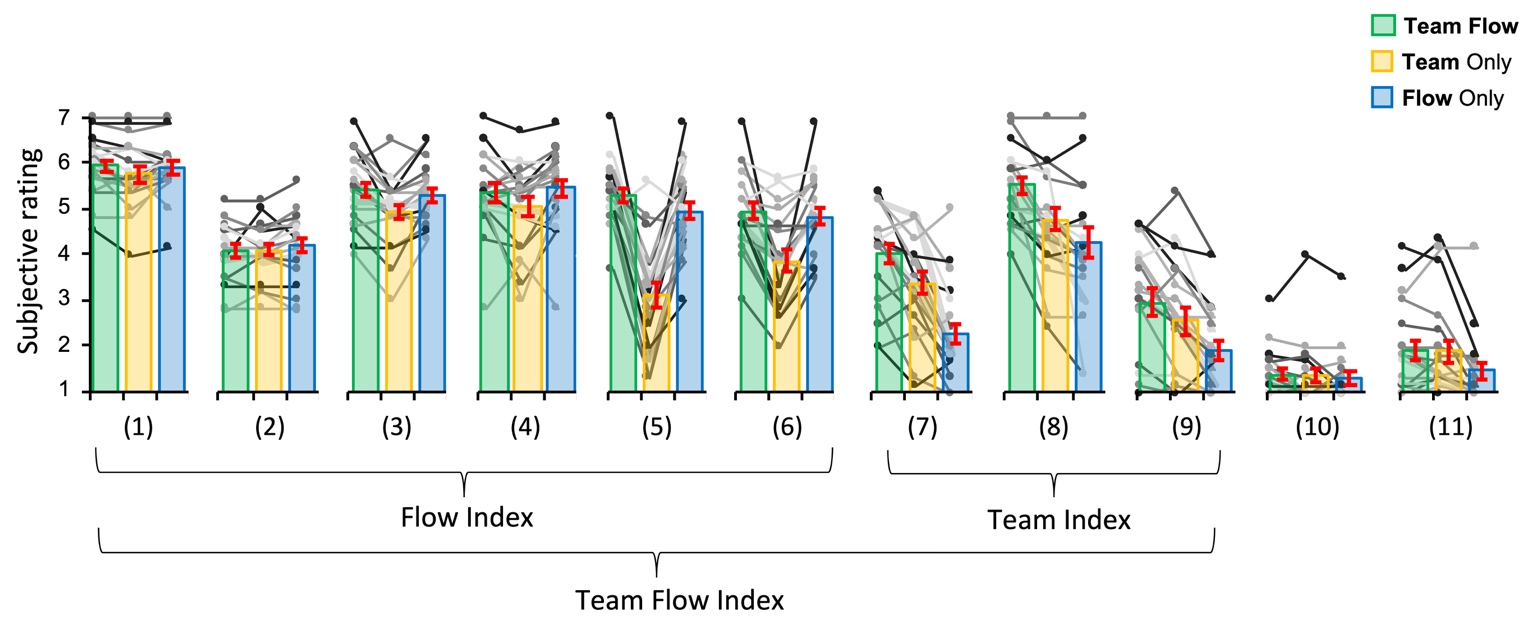

Supplement: Extended Data Figure 2-1 — Summary of subjective ratings for assessing the flow state and team interaction. The flow index was calculated by averaging responses for 1–6, the team index was calculated by averaging 7–9, and the team flow index was calculated by averaging 1–9. Psychometric ratings description: (1) “I had the necessary skill to play this trial successfully”; (2) “I will enjoy this trial more if it has less/more notes”; (3) “I felt in control while playing this trial”; (4) “I made correct movements automatically without thinking”; (5) “I love the feeling of this trial and want to play it again”; (6) “How time flies during this trial”; (7) “I was aware of the other player’s actions”; (8) “I felt like I was playing with the other person as a team”; (9) “I was coordinating my fingers with the other player’s fingers”; (10) “I felt like I was competing with the other player”; (11) “I was distracted by the other player’s actions” [for (2), rating 7 = more notes and 1 = less notes; for (6), rating 7 = fast and 1 = slow; for the rest, rating 7 = strongly agree and 1 = strongly disagree]. Error bars represent mean ± SEM; n = 20. Download Figure 2-1, TIF file. [file enu-eN-NWR-0133-21-s05.tif]

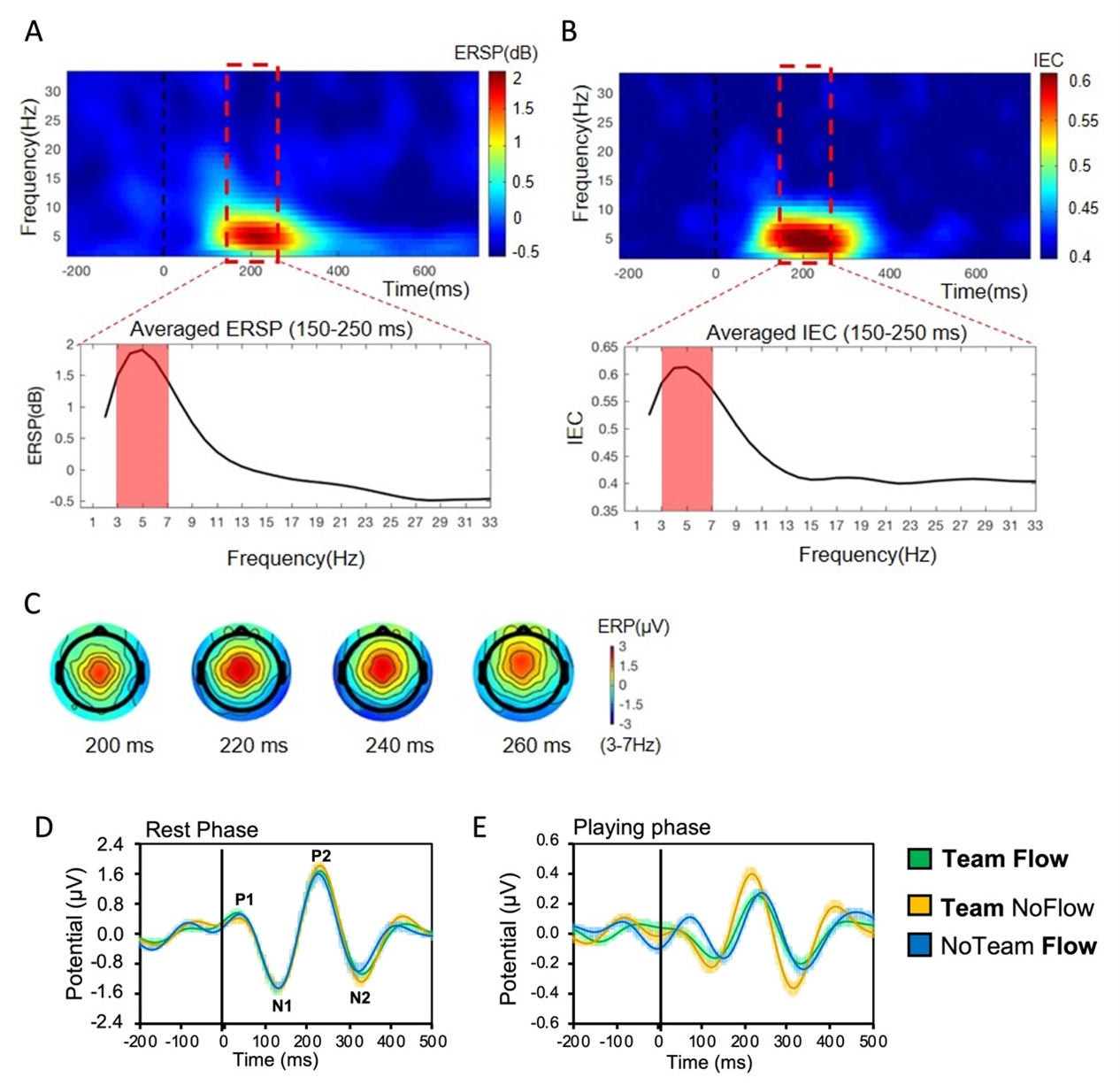

Supplement: Extended Data Figure 2-2 — A, B, Time-frequency analysis of the AEP locked to the task-irrelevant beep onsets, presented as the ERSP in A and the IEC analysis in B. Both ERSP and ITC showed changes in θ activity at 100–350 ms postonset (upper panel). An increase in θ activity (3–7 Hz) was prominent at 150–250 ms postonset (lower panel). C, Topographies of the event-related potential (ERP), bandpass-filtered in the θ range (3–7 Hz), at the indicated time points (ms) from the task-irrelevant beeps showing enhanced potential at the central channels. D, E, The potential, pass-filtered in the θ range (3–7 Hz), at central channels locked to the task-irrelevant beep onsets during the resting (D) and playing (E) phases. Download Figure 2-2, TIF file. [file enu-eN-NWR-0133-21-s06.tif]

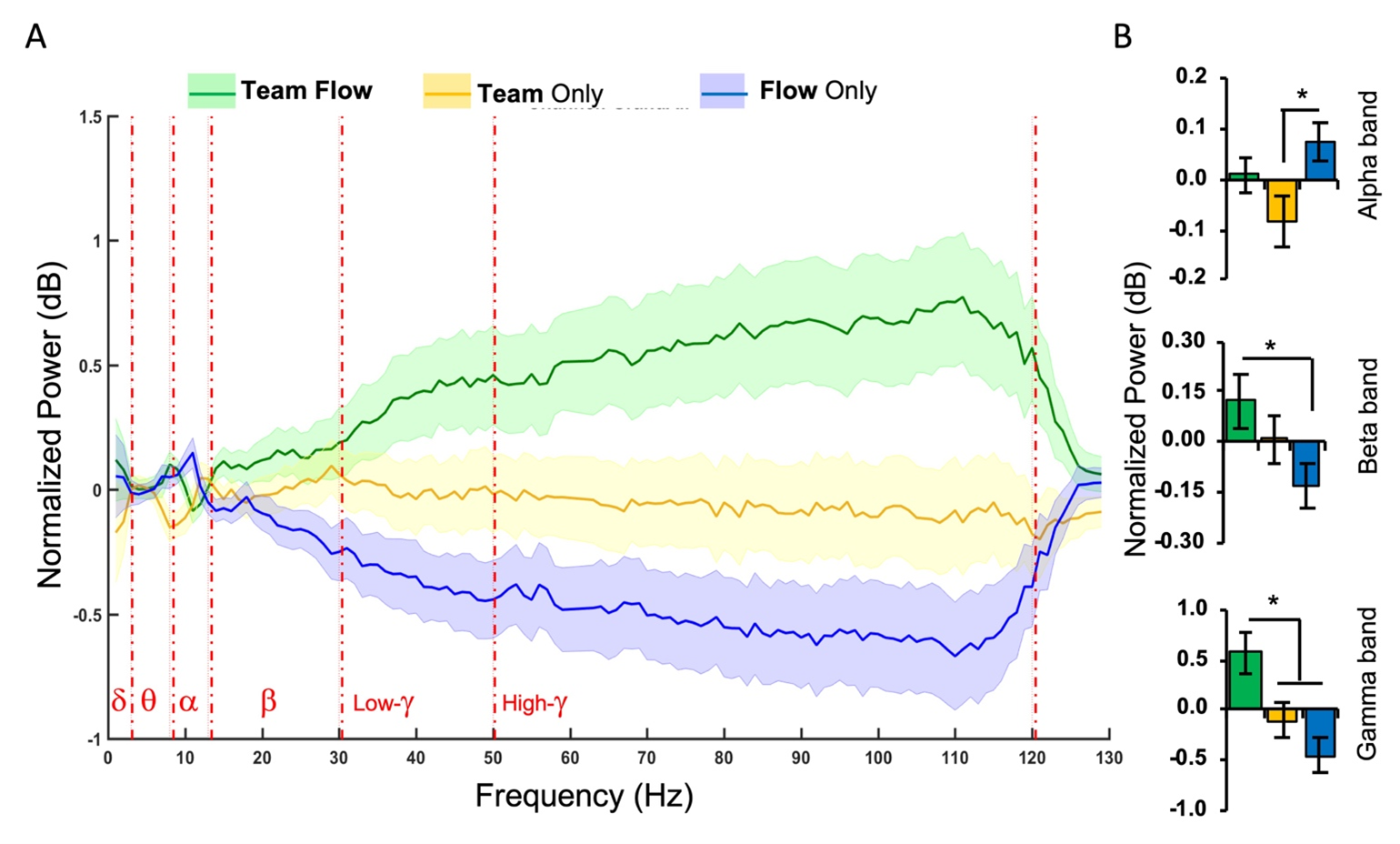

Supplement: Extended Data Figure 3-1 — A, The power difference spectral analysis for the three conditions grand averaged for all the 128 channels. B, Averaged individual power difference for the α (8–12 Hz), the β (13–30 Hz), and γ (31–120 Hz) frequency bands. One-way repeated measures ANOVA with Bonferroni post hoc test; *p < 0.05. Error bars represent mean ± SEM; n = 20. Download Figure 3-1, TIF file. [file enu-eN-NWR-0133-21-s07.tif]

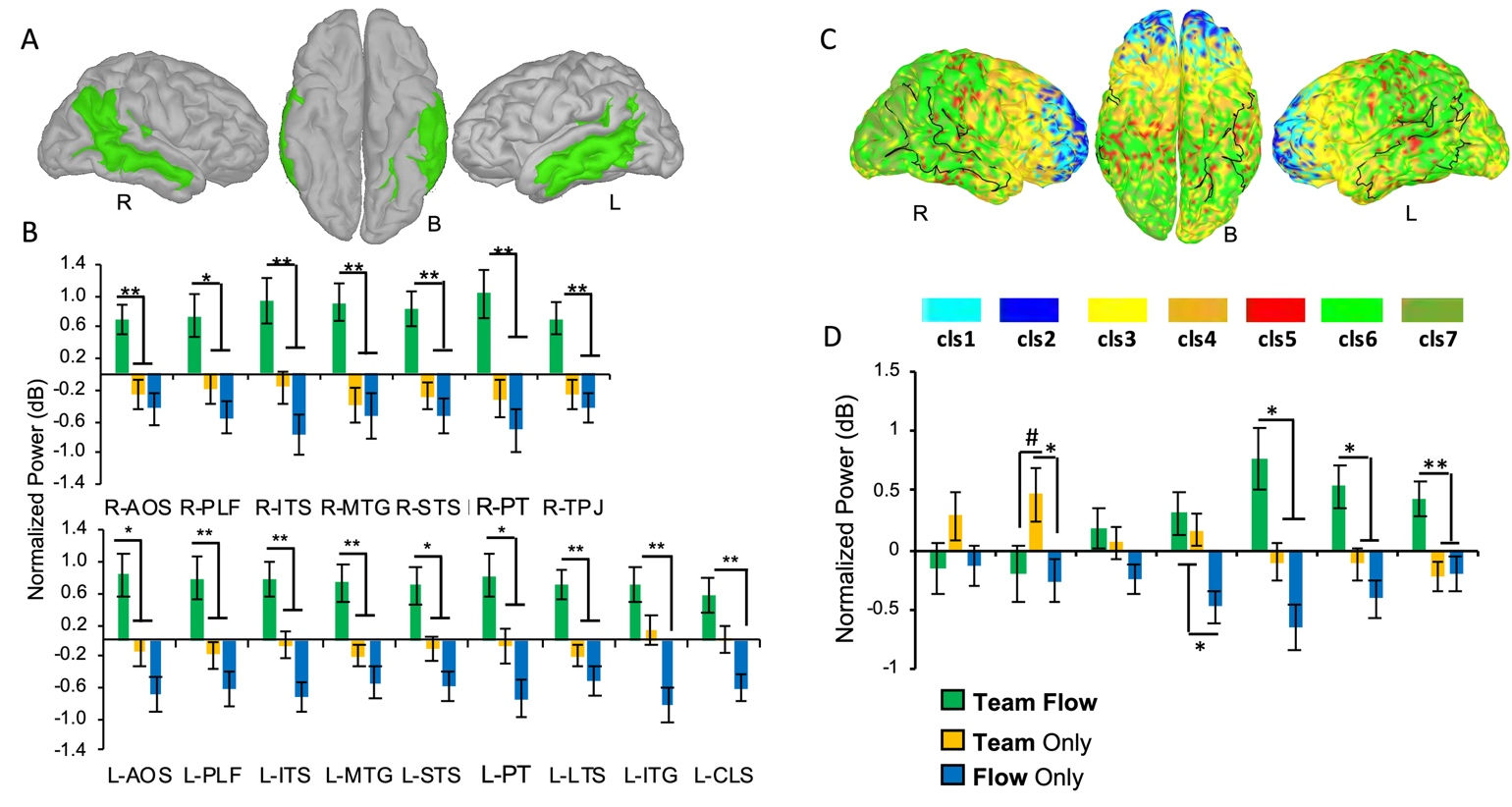

Supplement: Extended Data Figure 3-2 — Localization of the higher β-γ power during team flow. A, The brain regions (highlighted in green), as defined by the Destrieux atlas and showing significant β-γ normalized power difference across conditions. B, The average normalized β-γ power at the significant ROIs. Bonferroni-corrected critical value one-way ANOVA with Bonferroni post hoc test. C, D, Unsupervised hierarchical vertices clustering based on β-γ power difference between conditions. C, Clustered-vertices projected to a standard brain to visualize cluster localization. The black lines indicate the boundaries of the ROIs shown in B. D, The cluster-averaged normalized power of the β-γ power at each cluster. One-way repeated measures ANOVA with Tukey–Kramer’s post hoc test. Flow-related (cl 1–1–2), social-related (cls 3–4), or team flow-related (cls 5–7) clusters are indicated in the same color scheme as in D; *p < 0.05, **p < 0.01, #p = 0.077. Error bars represent mean ± SEM; n = 20. B, bottom view; R, right; L, left; AOS, anterior occipital sulcus; PLF, posterior lateral fissure; ITS, inferior temporal sulcus; MTG, middle temporal gyrus; STS, superior temporal sulcus; PT, superior plannar-temporal gyrus; TPJ, temporal parietal junction, LTS, lateral temporal sulcus; ITG, inferior temporal gyrus; CLS, collateral and lingual sulcus. Download Figure 3-2, TIF file. [file enu-eN-NWR-0133-21-s08.tif]

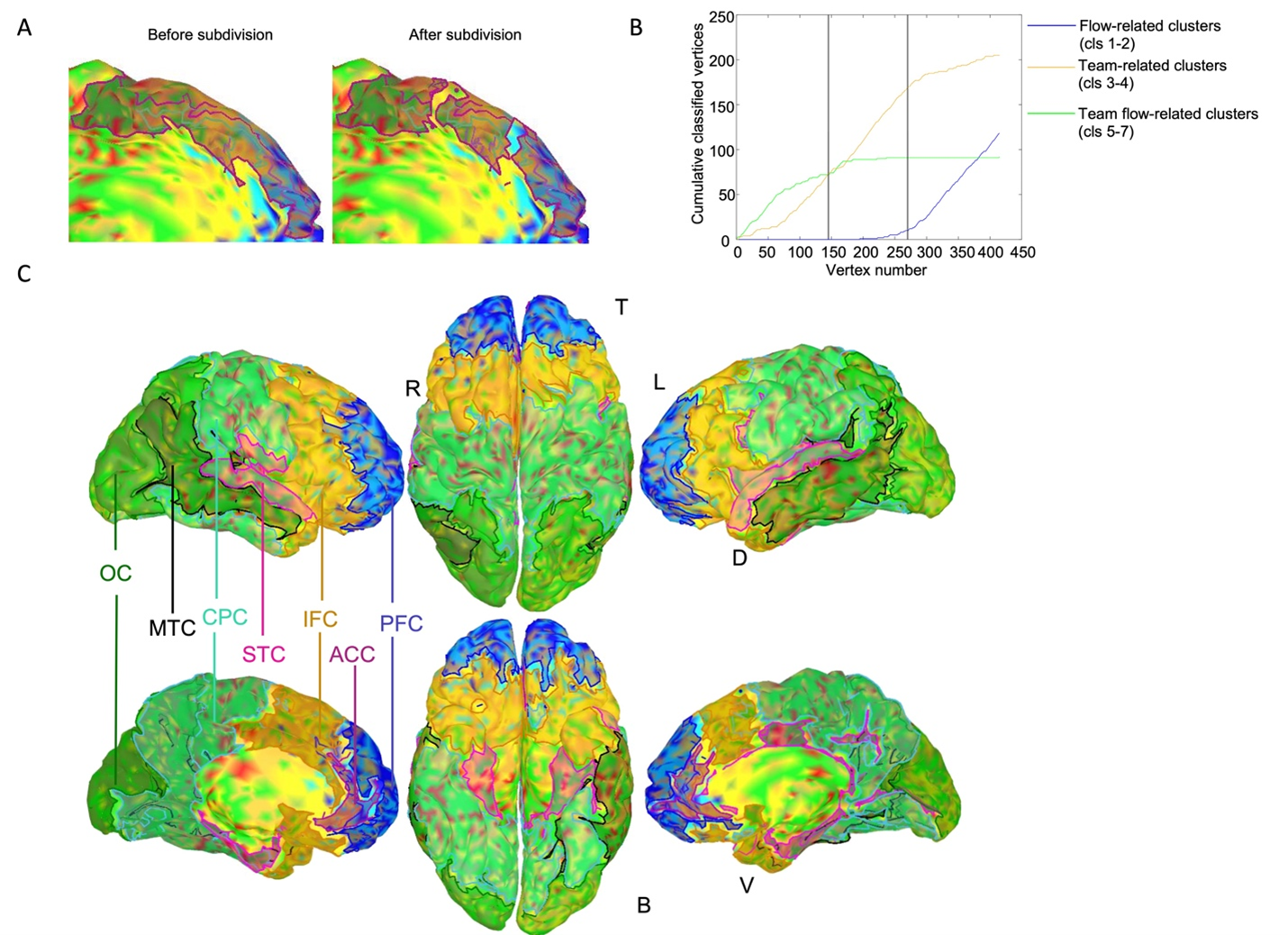

Supplement: Extended Data Figure 4-1 — Activity-dependent anatomically-defined grouping of ROIs (RGs). A, A medial view of the left superior frontal cortex (area inside the red boundary/transparent contour) before (left panel) and after (right panel) subdivision. B, The cumulative cluster composition curve for the left superior frontal cortex. The subdivision thresholds are shown as two vertical black lines subdividing this ROI into three subdivisions: flow-related subdivision (cls 1–2), team-related subdivision (cls 3–4), and team flow-related subdivision (cls 5–7). C, Transparent contours showing the brain regions which are also summarized in Extended Data Table 4-2. B, bottom; D, dorsal; L, left; R, right; T, top; V, ventral. Download Figure 4-1, TIF file. [file enu-eN-NWR-0133-21-s09.tif]

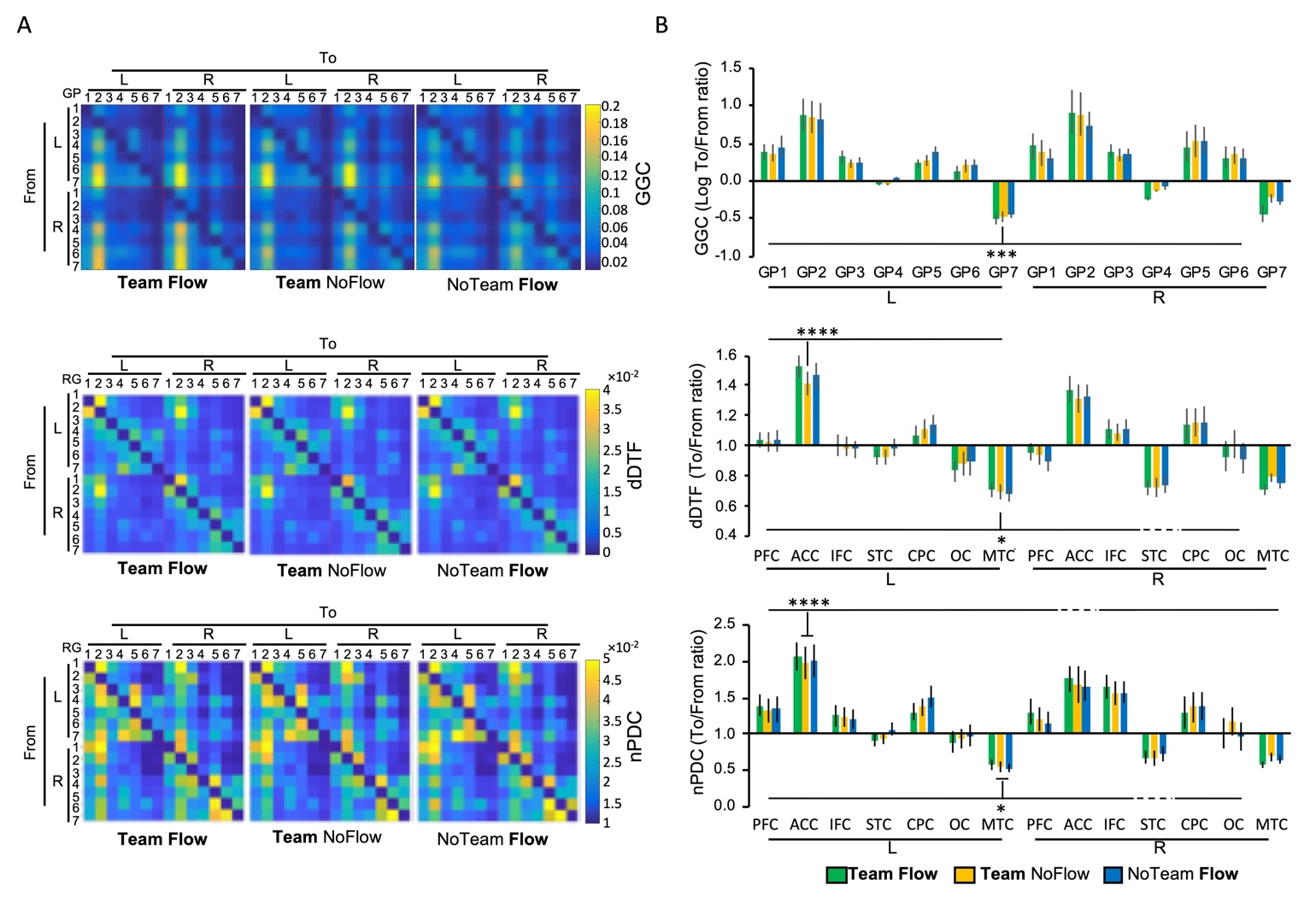

Supplement: Extended Data Figure 4-2 — Information causality analysis showing the MTC receives information from other brain regions. A, The mean causal interaction matrix for the brain regions (RGs). “To” indicates sending information; “from” indicates receiving information. The GGC (top), dDTF (middle), and the dDTF (bottom). L, left hemisphere; R, right hemisphere. B, The mean causal to/from ratio for GGC (top), dDTF (middle), and nPDC(bottom). In all GC measure, L-MTC (L-RG7) is a significant information receiver. Two-way repeated measures ANOVA with Tukey’s post hoc test; *p < 0.05, ***p < 0.001, ****p < 0.0001. Dashed line indicates p > 0.05. Error bars represent mean ± SEM; n = 20. Download Figure 4-2, TIF file. [file enu-eN-NWR-0133-21-s10.tif]
