# Supplementary material for: Team Flow Is a Unique Brain State Associated with Enhanced Information Integration and Interbrain Synchrony
Source: eNeuro. 2021 Oct 12;8(5):ENEURO.0133-21.2021. doi: 10.1523/ENEURO.0133-21.2021 (PMC8513532; doi:10.1523/ENEURO.0133-21.2021)
Supplement: Extended Data Figure 4-3 — Cluster composition (percentage) of the activity-dependent anatomically-defined groups (RGs). Download Figure 4-3, DOCX file. [file enu-eN-NWR-0133-21-s11.docx]

**Figure 4-3.**

**Cluster composition (percentage) of the activity-dependent anatomically-defined groups (RGs).**

|  | **cls1** | **cls2** | **cls6** | **cls7** | **cls3** | **cls4** | **cls5** | **Flow**  **-related** | **Team**  **-related** | **Team flow-related** |
| --- | --- | --- | --- | --- | --- | --- | --- | --- | --- | --- |
| **L-RG1** | 36.84 | 27.76 | 16.53 | 18.28 | 0.00 | 0.00 | 0.60 | **64.61** | 34.80 | 0.60 |
| **R-RG1** | 35.99 | 31.30 | 16.65 | 15.79 | 0.00 | 0.00 | 0.27 | **67.29** | 32.44 | 0.27 |
| **L-RG2** | 31.13 | 16.56 | 16.56 | 35.10 | 0.66 | 0.00 | 0.00 | **47.69** | 0.66 | **51.66** |
| **R-RG2** | 26.11 | 20.38 | 28.66 | 24.84 | 0.00 | 0.00 | 0.00 | **46.49** | **53.50** | 0.00 |
| **L-RG3** | 8.58 | 1.69 | 23.50 | 51.68 | 10.59 | 0.10 | 3.86 | 10.27 | **75.18** | 14.55 |
| **R-RG3** | 8.94 | 2.63 | 40.49 | 36.82 | 8.82 | 0.99 | 1.30 | 11.58 | **77.31** | 11.11 |
| **L-RG4** | 0.36 | 0.12 | 25.85 | 23.63 | 38.11 | 6.17 | 5.75 | 0.49 | **49.48** | **50.04** |
| **R-RG4** | 0.00 | 0.00 | 31.48 | 21.04 | 36.67 | 8.65 | 2.15 | 0.00 | **52.52** | **47.48** |
| **L-RG5** | 0.00 | 0.00 | 16.48 | 12.24 | 53.02 | 8.27 | 10.00 | 0.00 | 28.72 | **71.29** |
| **R-RG5** | 0.00 | 0.11 | 16.14 | 11.14 | 51.85 | 8.93 | 11.84 | 0.11 | 27.27 | **72.62** |
| **L-RG6** | 0.00 | 0.00 | 20.58 | 24.40 | 38.52 | 0.81 | 15.68 | 0.00 | **44.99** | **55.02** |
| **R-RG6** | 0.05 | 0.00 | 0.73 | 8.79 | 35.15 | 0.64 | 54.63 | 0.05 | 9.52 | **90.43** |
| **L-RG7** | 0.00 | 0.00 | 18.08 | 11.91 | 49.45 | 7.68 | 12.87 | 0.00 | 30.00 | **70.00** |
| **R-RG7** | 0.00 | 0.00 | 12.13 | 10.33 | 46.84 | 8.35 | 22.35 | 0.00 | 22.46 | **77.54** |
