# Supplementary material for: Team Flow Is a Unique Brain State Associated with Enhanced Information Integration and Interbrain Synchrony
Source: eNeuro. 2021 Oct 12;8(5):ENEURO.0133-21.2021. doi: 10.1523/ENEURO.0133-21.2021 (PMC8513532; doi:10.1523/ENEURO.0133-21.2021)
Supplement: Extended Data Figure 4-4 — Anatomical composition of the activity-dependent anatomically-defined groups (RGs). Download Figure 4-4, DOCX file. [file enu-eN-NWR-0133-21-s12.docx]

**Figure 4-4.**

**Anatomical composition of the activity-dependent anatomically-defined groups (RGs).**

| Abb. (RG#) | Anatomical ROIs (left hemisphere) | Anatomical ROIs (right hemisphere) |
| --- | --- | --- |
| PFC (RG1) | S_front_middle L; S_suborbital L; G&S_frontomargin L; G&S_transv_frontopol L | G&S_frontomargin R; S_front_middle R; G&S_transv_frontopol R |
| ACC (RG2) | G&S_cingul-Ant L | G&S_cingul-Ant R |
| IFC (RG3) | S_temporal_transverse L; S_orbital_lateral L; Pole_temporal L; Lat_Fis-ant-Vertical L; G&S_cingul-Mid-Ant L; S_orbital-H_Shaped L; S_circular_insula_ant L; G_front_inf-Opercular L; S_orbital_med-olfact L; G_temp_sup-Plan_polar L; S_front_inf L; G_subcallosal L; G_front_inf-Orbital L; G_front_inf-Triangul L; Lat_Fis-ant-Horizont L | G&S_cingul-Mid-Ant R; G&S_cingul-Mid-Post R; S_precentral-inf-part R; G_subcallosal R; S_suborbital R; G_temp_sup-G_T_transv R; G_insular_short R; S_circular_insula_ant R; S_circular_insula_sup R; S_orbital-H_Shaped R; G_temp_sup-Plan_polar R; G_front_inf-Opercular R; G_front_inf-Orbital R; S_front_inf R; Pole_temporal R; S_orbital_med-olfact R; S_orbital_lateral R; G_front_inf-Triangul R; Lat_Fis-ant-Horizont R; Lat_Fis-ant-Vertical R |
| STC (RG4) | S_circular_insula_sup L; S_precentral-inf-part L; S_subparietal L; G_oc-temp_med-Parahip L; G_insular_short L; G_temp_sup-Lateral L; S_collat_transv_ant L; G&S_cingul-Mid-Post L; G_Ins_lg&S_cent_ins L; S_pericallosal L; S_calcarine L; S_circular_insula_inf L | G_temp_sup-Lateral R; G&S_subcentral R; S_temporal_transverse R; G_Ins_lg&S_cent_ins R; G_oc-temp_med-Parahip R |
| CPC (RG5) | G_oc-temp_med-Lingual L; S_precentral-sup-part L; G&S_subcentral L; G_precuneus L; S_cingul-Marginalis L; G_oc-temp_lat-fusifor L; G_cingul-Post-dorsal L; G_temp_sup-G_T_transv L; G_precentral L; G&S_paracentral L; G_cingul-Post-ventral L; S_central L; G_pariet_inf-Supramar L; G_postcentral L; S_postcentral L; S_interm_prim-Jensen L | S_collat_transv_ant R; G_precentral R; S_central R; S_circular_insula_inf R; S_precentral-sup-part R; G_cingul-Post-ventral R; G_temporal_inf R; G_cingul-Post-dorsal R; S_oc-temp_med&Lingual R; G_postcentral R; S_oc-temp_lat R; G_oc-temp_lat-fusifor R; S_cingul-Marginalis R; G_oc-temp_med-Lingual R; G&S_paracentral R; S_postcentral R; S_subparietal R; G_precuneus R; G_pariet_inf-Supramar R; S_calcarine R; S_interm_prim-Jensen R |
| OC (RG6) | Pole_occipital L; S_oc_middle&Lunatus L; G_occipital_middle L; G_cuneus L; G&S_occipital_inf L; S_parieto_occipital L; G_occipital_sup L; S_collat_transv_post L; S_oc_sup&transversal L; G_pariet_inf-Angular L; G_parietal_sup L; S_intrapariet&P_trans L | S_oc_sup&transversal R; G_occipital_middle R; G_occipital_sup R; Pole_occipital R; G_cuneus R; S_oc_middle&Lunatus R; G&S_occipital_inf R; G_parietal_sup R; S_collat_transv_post R; S_parieto_occipital R; S_intrapariet&P_trans R |
| MTC (RG7) | G_temp_sup-Plan_tempo L; S_occipital_ant L; S_oc-temp_lat L; S_oc-temp_med&Lingual L; Lat_Fis-post L; S_temporal_sup L; S_temporal_inf L; G_temporal_inf L; G_temporal_middle L | G_pariet_inf-Angular R; S_occipital_ant R; S_temporal_sup R; G_temp_sup-Plan_tempo R; S_temporal_inf R; Lat_Fis-post R; G_temporal_middle R |
| Subdivided | G_orbital L; G_rectus L; G_front_middle L; S_front_sup L; G_front_sup L | G_rectus R; G_orbital R; G_front_middle R; S_front_sup R; G_front_sup R; S_pericallosal R |
